# Multipotent Adult Progenitor Cells Support Lymphatic Regeneration at Multiple Anatomical Levels during Wound Healing and Lymphedema

**DOI:** 10.1038/s41598-018-21610-8

**Published:** 2018-03-01

**Authors:** Manu Beerens, Xabier L. Aranguren, Benoit Hendrickx, Wouter Dheedene, Tom Dresselaers, Uwe Himmelreich, Catherine Verfaillie, Aernout Luttun

**Affiliations:** 1 Center for Molecular and Vascular Biology, Endothelial Cell Biology Unit, KU Leuven, Leuven, Belgium; 2 Cardiovascular Division, Brigham and Women’s Hospital, Harvard Medical School, Boston, USA; 3 Hematology and Cell Therapy Area, Clínica Universitaria and Foundation for Applied Medical Research, University of Navarra, Pamplona, Spain; 4 Department of Plastic and Reconstructive Surgery, University Hospital Brussels, Brussels, Belgium; 5 Biomedical MRI Unit, Department of Imaging and Pathology, KU Leuven, Leuven, Belgium; 6 Stem Cell Institute, KU Leuven, Leuven, Belgium; 7 Stem Cell Institute, Minneapolis, USA

## Abstract

Lymphatic capillary growth is an integral part of wound healing, yet, the combined effectiveness of stem/progenitor cells on lymphatic and blood vascular regeneration in wounds needs further exploration. Stem/progenitor cell transplantation also emerged as an approach to cure lymphedema, a condition caused by lymphatic system deficiency. While lymphedema treatment requires lymphatic system restoration from the capillary to the collector level, it remains undetermined whether stem/progenitor cells support a complex regenerative response across the entire anatomical spectrum of the system. Here, we demonstrate that, although multipotent adult progenitor cells (MAPCs) showed potential to differentiate down the lymphatic endothelial lineage, they mainly trophically supported lymphatic endothelial cell behaviour *in vitro*. *In vivo*, MAPC transplantation supported blood vessel and lymphatic capillary growth in wounds and restored lymph drainage across skin flaps by stimulating capillary and pre-collector vessel regeneration. Finally, human MAPCs mediated survival and functional reconnection of transplanted lymph nodes to the host lymphatic network by improving their (lymph)vascular supply and restoring collector vessels. Thus, MAPC transplantation represents a promising remedy for lymphatic system restoration at different anatomical levels and hence an appealing treatment for lymphedema. Furthermore, its combined efficacy on lymphatic and blood vascular growth is an important asset for wound healing.

## Introduction

Vertebrates have two vascular systems to distribute cells, gases and fluids. While blood vessels have been extensively studied, the lymphatic system gained significant interest by the growing notion that lymphatic dysfunction or hyperplasia is connected to cardiovascular disease, infection, cancer and obesity, the four principal healthcare challenges of this century^1^. In addition to blood vessel formation, adequate growth of lymphatic capillaries in the wound bed is essential during the normal wound healing response^2–4^. Indeed, lymphatics have been recently associated with granulation tissue formation, matrix remodelling and leukocyte trafficking in wound healing^2^. Defective healing of chronic wounds in diabetic patients can be attributed to a combined deficiency in blood vascular and lymphatic regenerative potential^4^. Furthermore, the importance and feasibility of incorporating lymphatic vessels in addition to blood vessels in dermo-epidermal skin grafts and substitutes was recently highlighted^5–7^. While stem/progenitor cells have been amply considered to promote wound healing, their effect on the lymphatic component during this process remains largely unexplored^8^.

Impaired lymphatic drainage results in lymphedema characterised by tissue swelling with risk for recurrent infections, ulcers, fibrosis and lipid accumulation^1^. While sometimes genetic in origin, lymphedema more frequently occurs secondary to damage to the lymphatic system, *e.g*., upon surgical (sentinel) lymph node removal and irradiation in cancer patients. Because of the lifelong course of the disease, lymphedema has a significant socio-economic burden. Current treatment is limited to conservative measures (*e.g*., compressive garment use) to alleviate symptoms rather than repairing the primary deficit. Importantly, lymphatic regeneration following lymphedema is likely more complex than during wound healing, since effective lymphedema treatment requires restoration of the lymphatic vasculature from the capillary to the collector level^9^.

Recently, innovative procedures have been developed for surgical or biological correction of lymphatic defects. Lymphatic grafting or lymphaticovenous anastomoses have met with variable clinical success. Vascularised lymph node transplantation became more feasible with the advent of supermicrosurgery, yet, the outcome seems inconsistent, likely because successful lymph node reintegration relies on spontaneous lymphangiogenesis^10,11^. Lymphangiogenic growth factor (*i.e*., vascular endothelial growth factor-C or VEGF-C) supplementation was introduced as adjuvant therapy to support lymph node transfer^9,11–13^. Despite relative success, growth factor use remains however associated with a risk for (systemic) side effects upon improper dosing^10^, unresponsiveness of endogenous lymphatic endothelial cells, the fact that growth factor monotherapy does not mimic the multifactorial physiology of lymphatic growth – potentially leading to dysfunctional lymphatic vessels^14^ – and the need for repeated administration given the short life-span and limited bioavailability through rapid diffusion of the growth factor^15^. Using stem/progenitor cells as a delivery system to locally and durably release a complement of (lymph)angiogenic growth factors may overcome these caveats. Furthermore, lymph vessels not only develop from venous endothelium but also by *de novo* incorporation of precursors of non-venous origin (‘lymphvasculogenesis’)^16–20^, suggesting that a similar process may occur during lymphatic regeneration in adults. Accordingly, stem/progenitor cells with, albeit limited, capacity to differentiate down the lymphatic endothelial cell lineage were found in cord/peripheral blood^21–25^ and bone marrow/adipose tissue (stroma)^22,23,25–31^. Multiple cell types have been tested in various animal models to evaluate their efficacy for lymphatic regeneration, including mesenchymal stem cells (reviewed in refs^32,33^), adipose tissue stem cells^31,32,34,35^, pluripotent stem cells^3^ and bone marrow-derived endothelial cell precursors^27^. However, no studies have provided evidence for their therapeutic potential to support restoration of the lymphatic vasculature from the capillary to the collector level, which is a prerequisite for effective lymphedema treatment^9^.

Bone marrow-derived MAPCs have multi-lineage differentiation potential, including the formation of arterial and venous endothelial cells^36,37^ and raise a robust angiogenic and arteriogenic response in ischaemic limbs^37,38^ and hearts, mainly by trophic support^39^. However, their ability to differentiate towards lymphatic endothelial cells and trophic contribution towards lymphatic regeneration remains undetermined. Here, we evaluated their potential to contribute to lymphatic growth in addition to blood vascular growth, in an array of lymphatic regeneration/growth models and report that they robustly contributed to restoration of a functional lymphatic system at the capillary and (pre-)collector level and mediated functional reintegration of transplanted lymph nodes.

## Results

### MAPCs have lymphvasculogenic and lymphangiogenic potential

When exposed to VEGF-A, mouse (m)MAPCs can be specified to arterial and human (h)MAPCs differentiate into arterial and venous endothelial cells^36,37^. Here, we investigated whether mouse and human MAPCs can differentiate down the lymphatic endothelial lineage under similar conditions. First, we confirmed that MAPCs gain general endothelial cell marker expression upon VEGF-A exposure (Supplementary Fig. S1a,b). In support of their lymphvasculogenic potential, *Prospero homeobox 1* (*Prox1)*, the lymphatic master switch, was significantly induced in MAPCs. This likely triggered expression of additional lymphatic genes (*i.e*., *Pdpn* and *Itg9a*), known to be upregulated by forced Prox1 expression (Supplementary Fig. S1c,d)^40^. A fraction (21 ± 6%) of VEGF-A-exposed MAPCs also expressed Lymphatic Vessel Endothelial Hyaluronan Receptor 1 (LYVE1; shown for mMAPCs; Supplementary Fig. S1e). Notably, lymphatic marker gene induction in hMAPCs was not improved by lymphangiogenic growth factor VEGF-C (shown for *LYVE1* in Supplementary Fig. S1f; *PROX1* fold-induction *versus* day 0 was also comparable upon exposure to VEGF-A, VEGF-C or a combination: 26 ± 10, 26 ± 14 and 26 ± 11, respectively; *n* = 4 independent differentiations).

**Figure 1:**
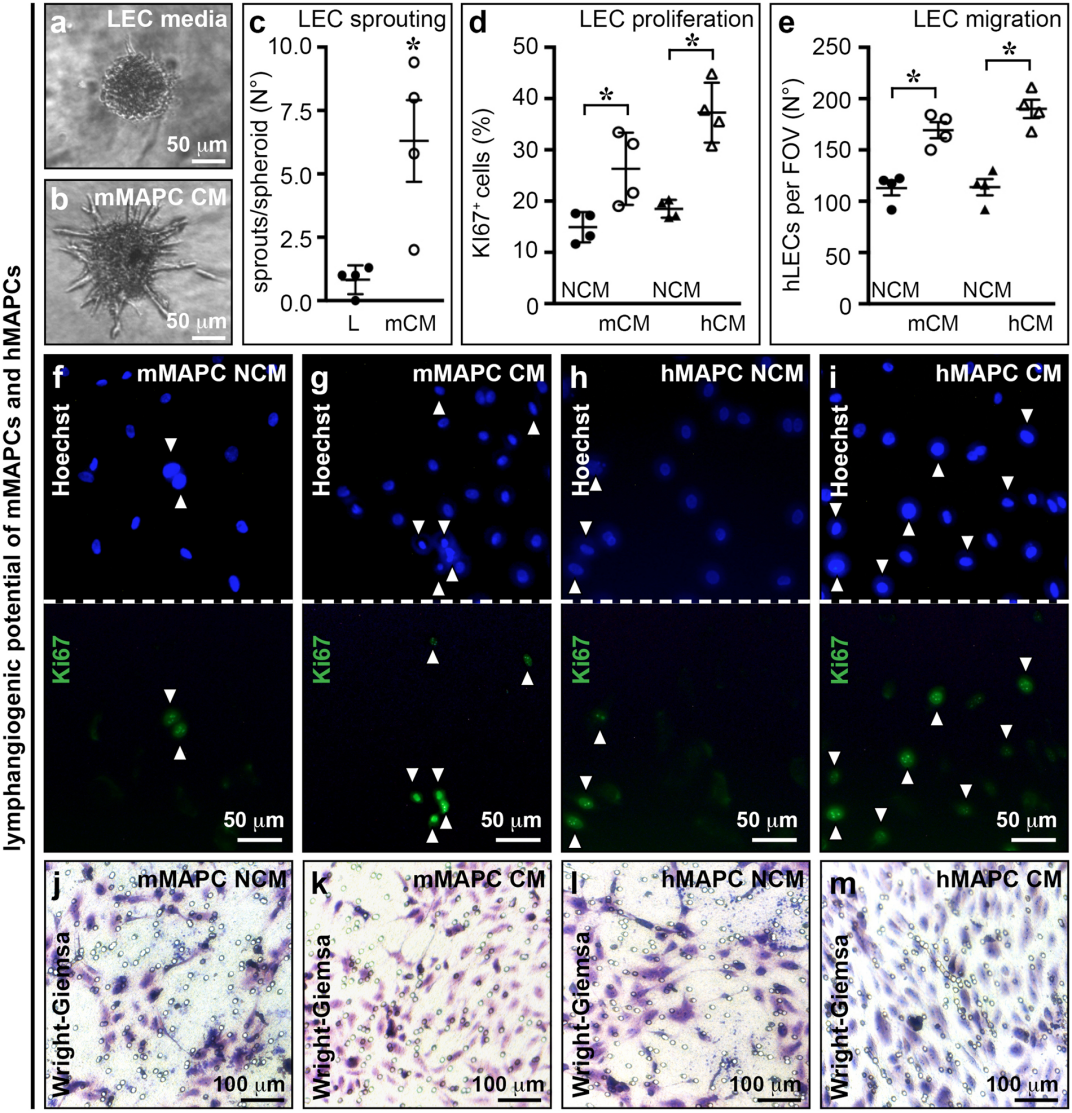
MAPCs have lymphangiogenic potential. **(a**–**c)** Images of human lymphatic endothelial cell (LEC) spheroids exposed to LEC media (*a*; ‘L’) or conditioned media from mMAPCs (‘mCM’; *b*), and corresponding quantification (*c*; *n* = 4; *P = 0.029 *versus* ‘L’ by Mann-Whitney-U test). (**d**–**m)** Images of LECs stained with proliferation marker Ki67 (in green in bottom half; top half shows corresponding field of view (FOV) stained with Hoechst in blue in the presence of non-conditioned mMAPC media (NCM; *f*), mMAPC-CM (*g*), hMAPC-NCM (*h*) or hMAPC-CM (*i*) or LECs migrated across the membrane of a transwell (revealed by Wright-Giemsa staining; *j*–*m*) and the corresponding quantifications (*d*: proliferation, expressed as % of Ki67^+^ cells, *n* = 4; *e*: migration, expressed as number of cells per FOV, *n* = 4; *P = 0.029 *versus* corresponding NCM condition by Mann-Whitney-U test).

In ischaemic limbs, MAPCs had a limited direct contribution to blood vascular endothelium. Hence, their effect was mainly due to a side-supply of angiogenic growth factors^37,38,41^. We reasoned that MAPCs could have an equally important trophic effect on lymphangiogenesis. Accordingly, 72 hour mMAPC- or hMAPC-conditioned media significantly stimulated lymphatic endothelial cell sprouting, proliferation and migration (Fig. 1a–m). To explore the factors potentially responsible for this lymphangiogenic effect, we first performed quantitative (q)RT-PCR for known lymphangiogenic growth factors and found that while mMAPCs and hMAPCs expressed *VEGF-A* and *angiopoietin-2* (*ANG-2*) to a similar extent, *VEGF-C* expression was only prominent in hMAPCs (Supplementary Fig. S2a). Furthermore, a more unbiased screen using antibody arrays on the (non-)conditioned media revealed that while mMAPCs and hMAPCs had a 62% overlap in their cytokine/growth factor secretion profile, hMAPCs not only secreted larger amounts, but also a broader complement of these factors, including VEGF-C (Supplementary Fig. S2b–d and Table S1).

### MAPCs support lymphatic capillary growth in healing wounds

**Figure 2:**
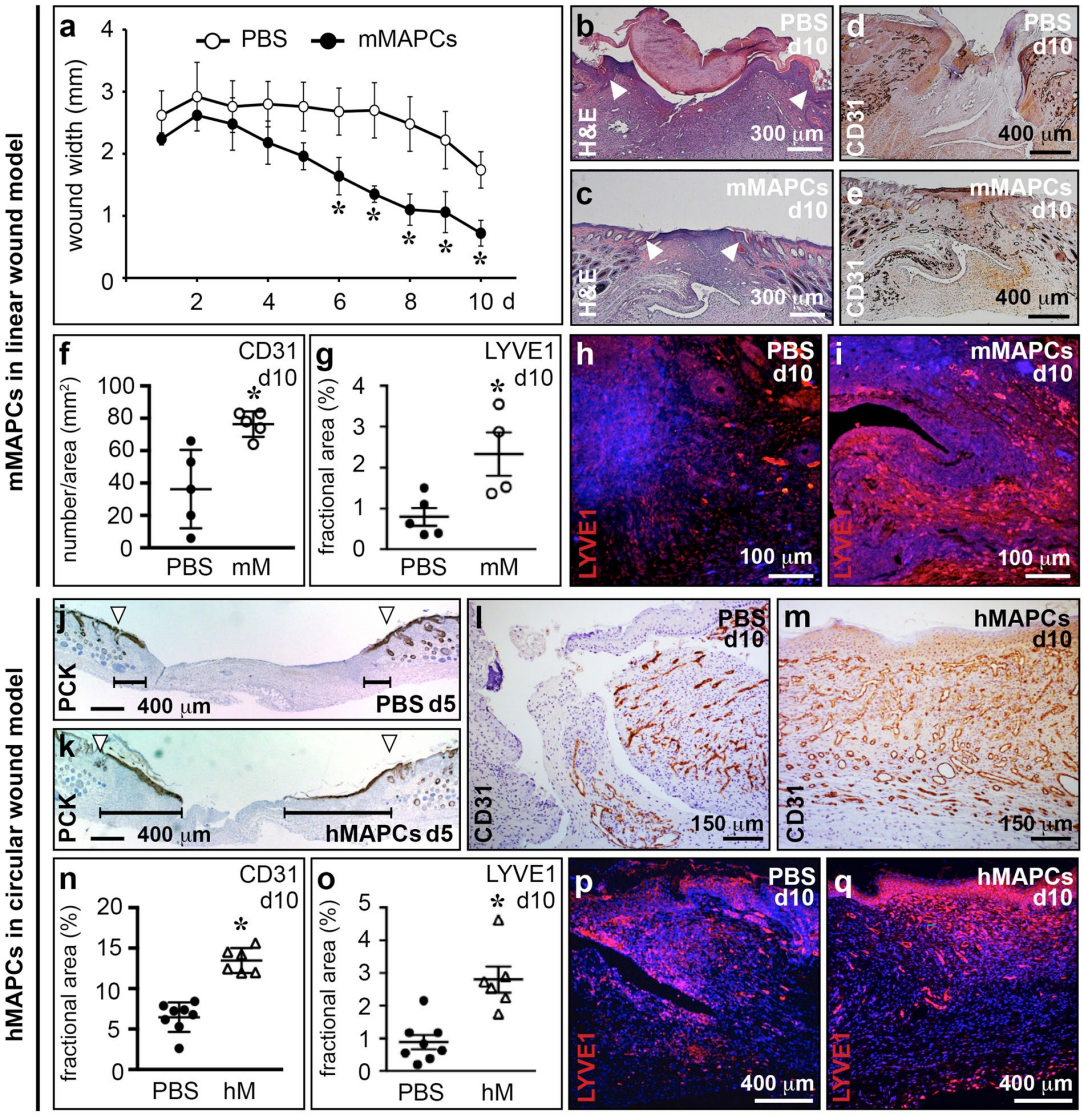
MAPCs stimulate blood vessel and lymphatic capillary growth in wounds. (**a**) Wound width in mice treated with PBS or mMAPCs (*n* = 5; *P < 0.05 *versus* PBS by repeated measures ANOVA with Fisher post-hoc test). (**b,c**) Representative pictures of cross-sections of 10 day (d)-old wounds from mice treated with PBS (*b*) or mMAPCs (*c*) stained with haematoxylin and eosin (H&E). Note the significantly smaller wound gap (the edges of which are indicated by arrowheads) in mMAPC-treated mice. (**d**–**f**) CD31-stained cross-sections of 10d-old wounds treated with PBS (*d*) or mMAPCs (*e*), and corresponding quantification (*f*; *n* = 5; *P = 0.008 *versus* PBS by unpaired two-tailed Student’s *t*-test). (**g**–**i**) LYVE1-stained (in red) cross-sections of 10d-old wounds treated with PBS (*h*) or mMAPCs (*i*), and corresponding quantification (*g*; *n* = 4–5; *P = 0.032 *versus* PBS by unpaired two-tailed Student’s *t*-test). (**j,k**) Cross-sections of wounds treated with PBS (*j*) or hMAPCs (*k*) 5d earlier, stained for pancytokeratin (PCK; arrowheads indicate wound borders, horizontal lines indicate distance covered by the epidermis). (**l**–**n**) CD31-stained cross-sections of wounds treated with PBS (*l*) or hMAPCs (*m*) 10d earlier, and corresponding quantification (*n* = 6–8; *P < 0.0001 *versus* PBS by unpaired two-tailed Student’s *t*-test). (**o**–**q**) LYVE1-stained (in red) cross-sections of 10d-old wounds after treatment with PBS (*p*) or hMAPCs (*q*), and corresponding quantification (*o*; *n* = 6–8; *P = 0.0007 *versus* PBS by unpaired two-tailed Student’s *t*-test). Haematoxylin or DAPI were used to reveal nuclei in *b-e*, *j-m* and *h*, *i*, *p*, *q*, respectively.

Wound healing, a physiological repair process, requires blood and lymphatic vessel growth^2,4^. Since MAPCs showed the capacity *in vitro* to give rise and offer trophic support to blood vascular^37,38^ and lymphatic endothelial cells (this study), we tested their potential to ameliorate wound healing. Transplantation of mMAPCs from mice ubiquitously expressing enhanced (e)GFP significantly accelerated wound closure and resulted in smaller scars (Fig. 2a–c + Supplementary Fig. S3a–c) compared to phosphate-buffered saline (PBS)-injection. While all mMAPC-injected wounds were completely re-epithelialised, 60% of PBS-treated wounds were only partially covered with neo-epidermis at 10 days. *In vivo* fluorescence imaging revealed that 4 days after injection, eGFP^+^ mMAPCs were in close vicinity to blood vessels growing towards the wound bed (Supplementary Fig. S3d,e). In accordance, mMAPC transplantation also boosted growth of CD31^+^ vessels in the wound centre by 2-fold, in limited part (2.8 ± 0.4% of engrafted cells) by direct contribution to CD31^+^ cells (Fig. 2d–f + Supplementary Fig. S3f). mMAPCs only occasionally contributed to differentiated lymphatic endothelial cells but significantly increased LYVE1^+^ or podoplanin^+^ lymphatic capillary growth by 3-fold (Fig. 2g–i + Supplementary Fig. S3g–j). The vast majority of LYVE1^+^ cells were lymphatic endothelial cells and not macrophage intermediates – previously suggested to contribute to lymphatic vessels in transplanted kidneys^26^ – since they did not express panleukocytic marker CD45 (Supplementary Fig. S3k).

Next, hMAPCs applied onto circular wounds accelerated wound closure (Supplementary Fig. S4a–c). Live imaging and cross-sections through the wound area showed their homogenous engraftment in the wound bed, with only occasional *in situ* differentiation to (lymphatic) endothelial cells, as shown by the intercalation of human-specific CD31^+^ cells in CD31^+^ host vessels and by the co-localisation of the hMAPC-derived vimentin signal and LYVE1, the latter both staining lymphatic endothelial cells from human donor and mouse recipient origin (Supplementary Fig. S4d–g). hMAPCs accelerated epithelial coverage (% coverage at 5 days: 46 ± 5 in hMAPC-treated *versus* 7 ± 2 in vehicle-treated wounds; *n* = 6, *P* < 0.0001 by unpaired two-tailed Student’s *t*-test; Fig. 2j,k), likely by increasing keratinocyte numbers in the advancing epithelial tongues (number of keratinocytes/mm at 5 days: 1,160 ± 87 in hMAPC-treated *versus* 440 ± 30 in PBS-treated wounds; *n* = 6, *P* < 0.0001 by unpaired two-tailed Student’s *t*-test) and increased granulation tissue formation by two-fold (Supplementary Fig. S4h–j). All wounds were completely re-epithelialised in hMAPC-treated mice *versus* only 46% of PBS-treated mice and hMAPC-treated wounds showed improved collagen remodelling at 10 days (determined by the % organised red-birefringent collagen; Supplementary Fig. S4k–m). hMAPC transplantation improved wound vascularisation by about 2-fold at 10 days (determined by the % CD31^+^ area in the entire wound; Fig. 2l–n). hMAPCs significantly boosted lymphangiogenesis as evidenced by the 3-fold increased LYVE1^+^ fractional area and the 2-fold increase in podoplanin^+^ vessel density at 10 days (Fig. 2o–q + Supplementary Fig. S4n–p). Double immunofluorescence staining for Prox1 and smooth muscle α-actin (αSMA) revealed that the vast majority (97 ± 2%) of lymphatic vessels in granulation tissue at 10 days were capillaries devoid of αSMA coverage.

### MAPCs support lymphatic capillary and pre-collector restoration in elevated skin flaps

**Figure 3:**
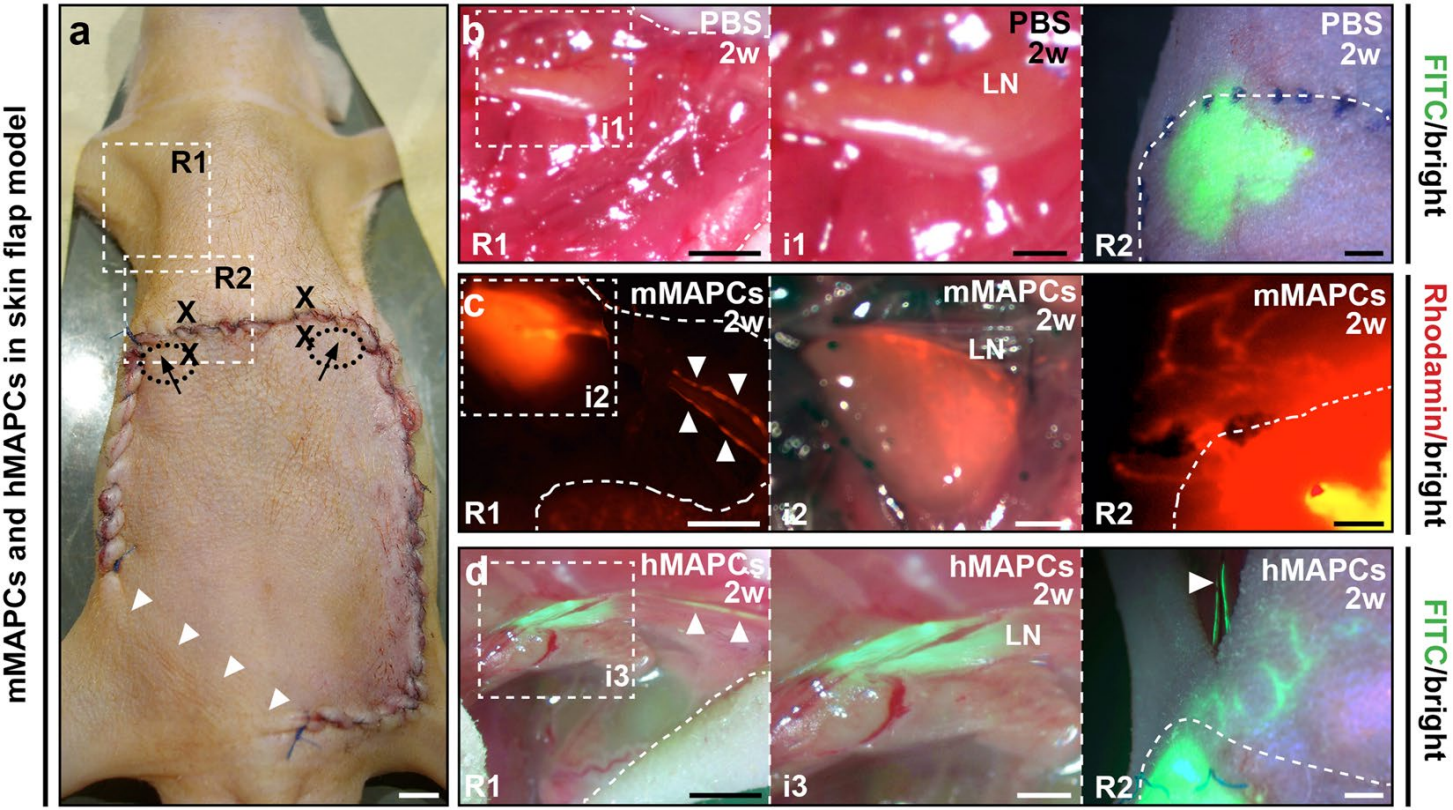
MAPCs restore lymph drainage across a severed lymphatic network. (**a**) Image displaying the skin flap model. R1/R2 indicate areas from which images in panel *b*-*d* are shown. Arrows/‘X’ indicate injection spots of fluorescently-labelled dextran for lymphangiography or MAPCs/PBS, respectively, and arrowheads show the area through which blood supply to the skin flap is preserved. (**b**–**d**) Merged pictures of bright field/fluorescence images 15 minutes after injection of dextran (FITC (green)-labelled in *b*,*d* or Rhodamin-B-(red)-labelled in *c*) of regions R1 (left panels; and enlarged image of the corresponding inset (i) in the middle panels) and R2 (right panels) of mice injected 2 weeks (w) earlier with PBS (*b*), mMAPCs (*c*) or hMAPCs (*d*). Arrowheads indicate filled afferent lymphatic vessels. LN: lymph node. Dashed lines delineate border of the opened skin in R1 or the flap border in R2.

**Figure 4:**
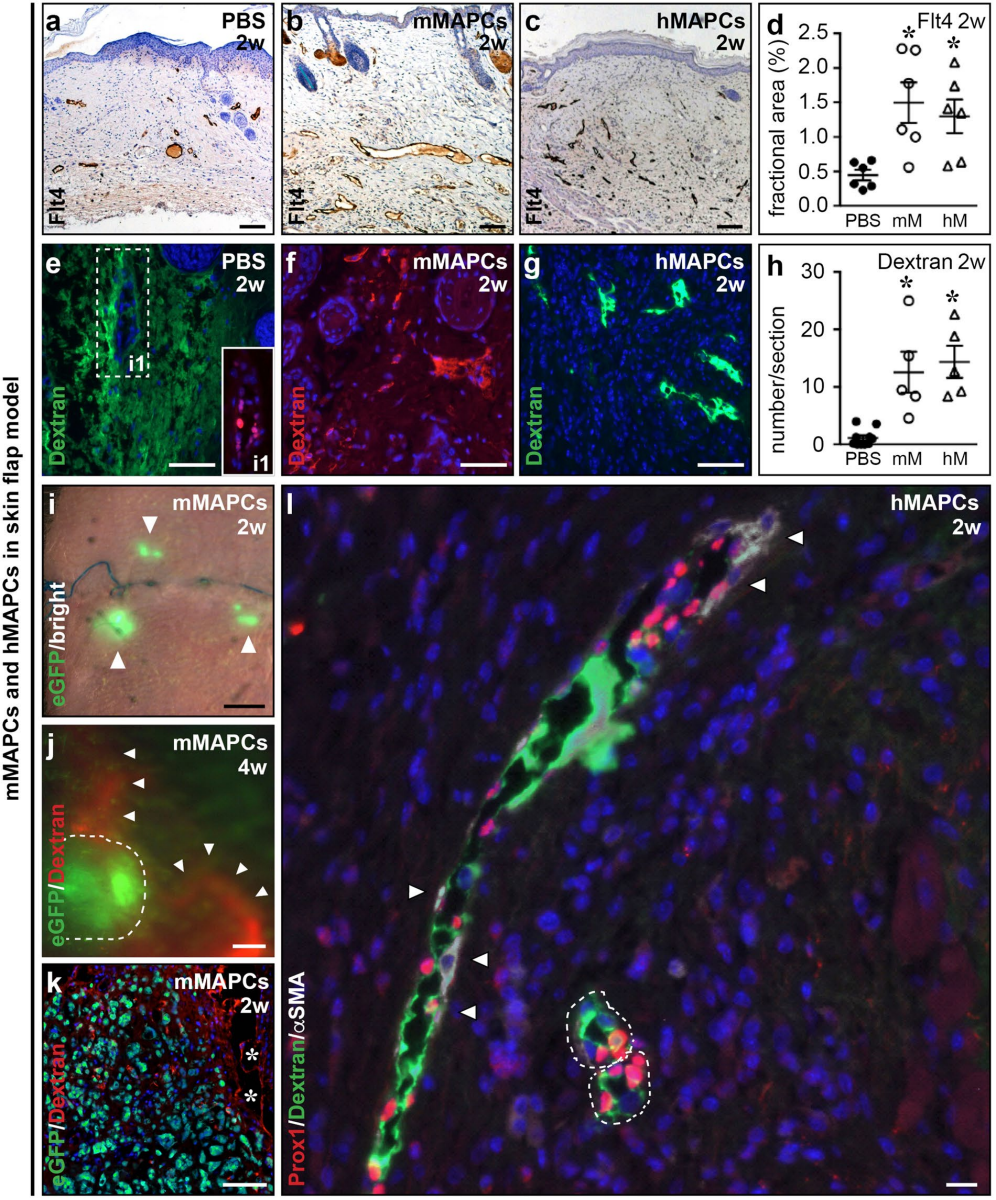
MAPCs restore lymphatic capillaries and pre-collectors. (**a**–**d**) Flt4-stained wound cross-sections from PBS (*a*), mMAPC (‘mM’; *b*) or hMAPC-treated (‘hM’; *c*) mice, and corresponding quantification (*d*; *n* = 6; *P* = 0.0074 by Kruskal-Wallis test; *P < 0.05 *versus* PBS by Dunn’s post-hoc test). (**e**–**h**) Wound cross-sections from PBS (*e*), mMAPC (*f*) or hMAPC-treated (*g*) mice revealing functional (dextran (red or green)-perfused) lymphatics in cell-treated mice, and corresponding quantification (*h*; *n* = 5–10; *P* < 0.0001 by Kruskal-Wallis test; *P < 0.05 *versus* PBS by Dunn’s post-hoc test). Inset (i1) in *e* shows corresponding Prox1-stained (in red) region. Note diffuse fluorescence signal in *e* representing FITC-dextran that failed to be drained. (**i**) Merged bright field/fluorescence image of the wound transplanted with eGFP^+^ mMAPCs (in green; indicated by arrowheads) 2 w earlier. (**j**) Merged green/red fluorescence images of the wound transplanted with eGFP^+^ mMAPCs (circled by dashed line) 4 w earlier. Note Rhodamin-dextran-filled lymphatic vessels (red; indicated by arrowheads) in the vicinity of transplanted cells. (**k**) Cross-section through the wound, revealing transplanted eGFP^+^ mMAPCs (in green) adjacent to functional (red Rhodamin-dextran-filled) lymphatics (asterisks). (**l**) Merged picture of green (FITC-labelled dextran), red (Prox1) and far-red (αSMA) fluorescence images of a wound transplanted with hMAPCs 2 w earlier, revealing a functional sparsely αSMA-coated (indicated by arrowheads) Prox1^+^ lymphatic pre-collector and two functional Prox1^+^/αSMA^−^ lymphatic capillaries (circled by white dashed lines). Haematoxylin or DAPI were used to reveal nuclei in *a–*c and *e–g*, *k*, *l*, respectively.

To test and compare the potential of mMAPCs and hMAPCs to functionally restore lymph flow through repair of a discontinued draining lymphatic system of the skin, we disrupted lymph drainage to the axillary lymph nodes by making a full-thickness skin incision in the abdomen (Fig. 3a)^42^. This intervention abrogated lymph drainage in the majority (7 out of 10) of PBS-treated animals shown by the lack of fluorescent dye crossing the wound border 2 weeks following skin incision (Fig. 3b; Table 1). MAPC transplantation almost completely (in 5 out of 6 and 6 out of 6 cases for mMAPC- or hMAPC-treated mice, respectively) restored drainage across this border (Fig. 3c,d; Table 1). While drainage to axillary lymph nodes was only obtained in 1 out of 10 PBS-injected mice, 3 out of 6 mMAPC-injected and 6 out of 6 hMAPC-injected mice showed lymph node drainage after 2 weeks. In a second set of mice injected with PBS or mMAPCs, fluorescent dye crossed the wound border in 5 out of 5 mMAPC-treated mice and lymph node drainage was restored in 4 out of 5, while there was no restoration of drainage across the wound border and into the axillary lymph nodes in any of the PBS-injected mice 4 weeks after skin incision (Table 1). Histological analysis of the skin wound area around the transplantation sites revealed that, in addition to a 1.8-fold expansion of CD31^+^ blood vessels (Supplementary Fig. S5a–d), MAPC-injected mice had a ~two-three-fold increase in fms-like tyrosine kinase (Flt)4^+^ (VEGFR3^+^) and LYVE1^+^ fractional area in the wound borders (Fig. 4a–d + Supplementary Fig. S5e–h, respectively) 2 weeks after skin incision. The average number of functional (dextran-filled) lymphatic vessels per cross-section around the incision at 2 weeks was significantly increased by MAPC injection (Fig. 4e–h). Notably, some mMAPCs persisted until 2–4 weeks and lodged in the vicinity of draining lymphatic vessels (Fig. 4i–k). Compared to the wound healing models, deep sparsely αSMA-coated Prox1^+^ pre-collector vessels were more frequently observed here (a representative example is shown in Fig. 4l), yet the majority (67 ± 5%) of skin lymphatics was still devoid of αSMA coating. Nevertheless, in addition to expanding the LYVE1^+^ capillary network, hMAPC transplantation increased the number of draining pre-collectors by 3-fold after 2 weeks (Table 1).

**Table 1:** Lymphatic function/anatomy in skin flap and lymph node transplantation models.

**Skin flap model**												
**Parameter/treatment**			**PBS**				**mMAPCs**				**hMAPCs**	
*Week post-op*			*2*		*4*		*2*		*4*		*2*	
Wound border crossing (%)			30.0		0.0		83.3		100.0		100.0	
Lymph node filling (%)			10.0		0.0		50.0		80.0		100.0	
Dextran^+^Prox1^+^αSMA^+^ pre- collectors (average number per cross-section)			3 ± 1		ND		ND		ND		10 ± 3^a^	
**Lymph node transplantation model**												
**Parameter/treatment**	**PBS**				**hMAPC1**				**hMAPC2**			
***Week post-op***	***4***	***8***		***16***	***4***	***8***		***16***	***4***	***8***		***16***
Survival (%)	100.0	83.0		50.0	100.0	100.0		100.0	100.0	100.0		100.0
Size (mm^2^x10)	7.4 ± 0.2	2.5 ± 1.0		3.5 ± 2.4	6.4 ± 1.3	12.0 ± 1.8^a^		10.8 ± 1.2^b^	ND	ND		ND
Branching (%)	0.0	0.0		0.0	100.0	100.0		100.0	100.0	100.0		100.0
Filling (%)	0.0	0.0		0.0	0.0	33.3		62.5	0.0	37.5		50.0

Data represent fraction of mice revealing the (functional) feature mentioned in the left column or mean ± s.e.m. (skin flap model: PBS: *n* = 10 for each time point; mMAPCs: *n* = 6 for 2 weeks and *n* = 5 for 4 weeks; hMAPCs: *n* = 6; lymph node transplantation model: PBS: *n* = 10, 6 and 6 for 4, 8 and 16 weeks, respectively; hMAPC1: *n* = 10, 6 and 8 for 4, 8 and 16 weeks, respectively; hMAPC2: *n* = 6, 8 and 4 for 4, 8 and 16 weeks, respectively). post-op: post-operation; ND, not determined. ^a^*P* = 0.022 and ^b^*P* = 0.019 *versus* corresponding PBS condition by Mann-Whitney-U test.

### hMAPCs reconnect transplanted lymph nodes to the host lymphatic network

**Figure 5:**
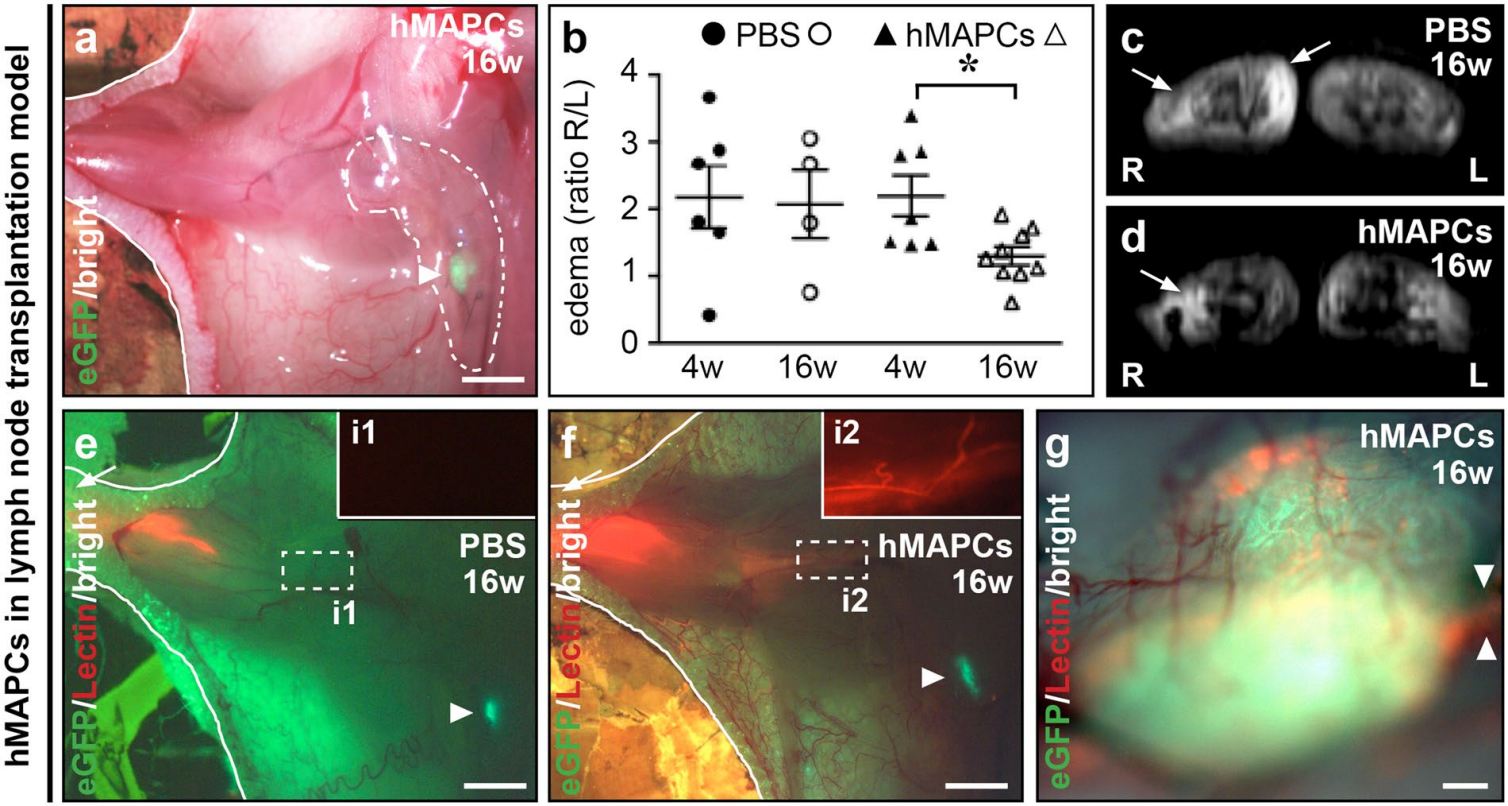
hMAPCs support functional reconnection of transplanted lymph nodes. (**a**) Merged bright field/fluorescence image of right axillary region 16 weeks (w) post-transplantation of an eGFP^+^ lymph node (LN; green; arrowhead) and treatment with Matrigel containing hMAPCs (dashed and full white lines indicate Matrigel-covered area and open skin border, respectively). (**b**) Edema extent in right upper limb (shown as rigth/left ratio in arbitrary units) 4 w or 16 w after LN transplantation and treatment with Matrigel containing PBS or hMAPCs. *n* = 4–9; *P = 0.011 *versus* 4 w by unpaired two-tailed Student’s *t*-test. (**c,d**) T_2_-weighted MR images of antebrachial regions 16 w after LN transplantation and treatment with Matrigel containing PBS (*c*) or hMAPCs (*d*). Hyperintense areas (arrows) indicate fluid accumulation. L: left; R: right. (**e,f**) Merged bright field/fluorescence image of right axillary region 16 w post-transplantation of an eGFP^+^ LN (green; arrowhead) and treatment with Matrigel containing PBS (*e*) or hMAPCs (*f*). Insets (i1,2; red channel only) zoom in on boxed areas in *e,f*. Note significantly improved drainage of Rhodamin-labelled (red) lectin in hMAPC-treated mice (arrow and white lines indicate lymphangiography injection spot and open skin border, respectively). (**g**) Merged bright field/fluorescence image zooming in on an eGFP^+^ (in green) LN transplanted in a mouse treated with Matrigel containing hMAPCs 16 w earlier, revealing uptake of red Rhodamin-labelled lectin. Arrowheads indicate connecting lymph vessel.

Thus far, we showed that MAPC transplantation increased lymphangiogenesis and reinstated lymphatic drainage mainly by boosting restoration of small caliber lymphatic vessels. However, the underlying problem of secondary lymphedema most often relates to damaged lymph nodes and large lymphatic collectors to which the lymphatic capillaries and pre-collectors normally connect. Hence, an appropriate remedy must equally imply restoration of lymphatic collectors. We applied a stringent model in which axillary lymph nodes and their surrounding lymphatic (collector) network were surgically ablated, such that drainage of a lymph node transplanted in this area becomes critically dependent on restoration of lymphatic collectors and their reconnection to the host lymphatic network^9^. To test the potential of hMAPCs, we applied them in Matrigel around a transplanted lymph node derived from mice ubiquitously expressing DsRed or eGFP in the right axillary cavity (Fig. 5a). Transplantation of the lymph node alone (and covering it with Matrigel containing PBS) failed to resolve inflammation-induced edema in the right upper limb, evident from interstitial fluid accumulation measured by magnetic resonance imaging (MRI) 4 and 16 weeks after surgery upon challenge of the paw with mustard oil – an inflammatory agent (Fig. 5b,c + Supplementary Fig. S6a). At 16 weeks, fluid accumulation was significantly less prominent upon application of hMAPCs around the transplanted lymph node, suggesting functional restoration of lymph drainage from the front paw to the axillary region (Fig. 5b,d + Supplementary Fig. S6b). Indeed, lymphangiography revealed that lymph fluid drainage was significantly improved in hMAPC-treated mice and that the injected fluorescent dye reached and filled the transplanted lymph node in ~35% and 50–60% of hMAPC-treated mice, 8 and 16 weeks post-transplantation, respectively, a result that was reproduced with two hMAPC populations and not at all in PBS-treated mice (Fig. 5e–g + Supplementary Fig. S6c; Table 1). This suggested that hMAPC transplantation functionally reconnected the transplanted lymph node to the host lymphatic network. Notably, while all lymph nodes implanted along with hMAPCs persisted, half of them could not be traced in PBS-injected mice at 16 weeks, suggesting a positive effect of hMAPC transplantation on lymph node survival (Table 1). Moreover, unlike in hMAPC-treated mice, the mean size of the engrafted lymph nodes was decreased in PBS-treated mice (Table 1).

**Figure 6:**
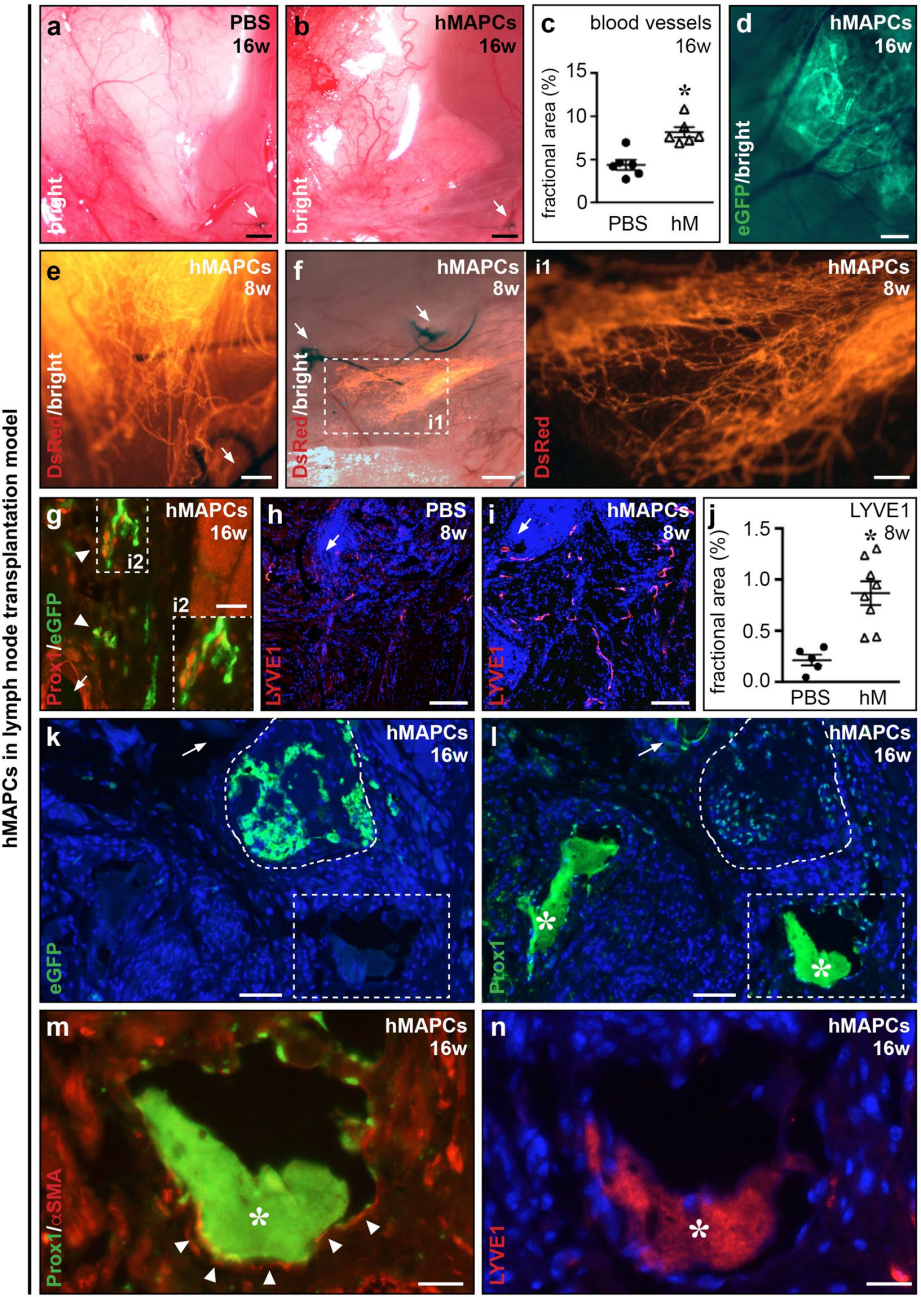
hMAPCs support lymph node survival and reconnection through blood vessel and lymphatic collector regeneration. (**a**–**c**) Bright field images of blood vascular network leading up to the transplanted lymph node (LN) of mice treated with Matrigel containing PBS (*a*) or hMAPCs (‘hM’; *b*) 16 weeks (w) earlier, and corresponding quantification (*c*; *n* = 6; *P = 0.0011 *versus* PBS by unpaired two-tailed Student’s *t*-test). (**d**) Merged bright field/fluorescence image of an eGFP^+^ (in green) LN 16 w post-transplantion and treatment with Matrigel containing hMAPCs revealing that the LN is irrigated by numerous blood vessels. (**e,f**) Merged bright field and fluorescence images zooming in on a DsRed^+^ (in red) LN 8 w post-transplantion and treatment with Matrigel containing hMAPCs revealing extensive LN vascular network branching. Inset (i1) corresponds to boxed area in *f*. (**g**) Merged immunofluorescence image of a Prox1 (in red)/eGFP (in green)-stained section in a mouse treated with Matrigel + hMAPCs 16 w earlier revealing that part of the branches are lymphatic in nature (Prox1^+^, indicated by arrowheads). Inset (i2) corresponds to boxed area in *g*. (**h**–**j**) LYVE1-stained (in red) cross-sections of PBS (*h*) or hMAPC-treated (‘hM’; *i*) mice in the area around the sutures at 8 w after LN transplantation and corresponding quantification (*j*; *n* = 5–8; *P = 0.0014 by unpaired two-tailed Student’s *t*-test *versus* PBS). (**k**–**n**) Fluorescence images of the area around the transplanted eGFP^+^ LN (in green; lined by a dashed line in *k*; adjacent Prox1 (in green)-stained section is shown in *l*; Prox1 (in green)/αSMA (in red) (arrowheads; double staining in *m* zooms in on the boxed area in *k*,*l*; and *n* represents the same area on an adjacent LYVE1 (in red)-stained cross-section) revealing Prox1^+^αSMA^+^LYVE1^-^ lymphatic collector vessels in mice treated 16 w earlier with Matrigel containing hMAPCs. Asterisks in *l-n* indicate lymph (which artefactually fluoresces upon exposure to tyramide-based amplification which was used for Prox1 and LYVE1 staining). White arrows in *a, b, e-i, k, l* indicate sutures used to fix the transplanted LN.

Inspection of the skin area leading up to the transplanted lymph node revealed a two-fold more elaborate blood vascular network in hMAPC-treated mice (Fig. 6a–c) with significantly more blood vessels in the immediate surrounding of the lymph nodes, compared to PBS-injected mice (Fig. 6d + Supplementary Fig. S6d,e). Some hMAPCs persisted until 16 weeks and were found in the vicinity of the transplanted lymph node (Supplementary Fig. S6f). All transplanted lymph nodes in hMAPC-treated mice showed signs of (outward) branching of their internal (lymph)vascular network from 4 weeks onwards, while this was never observed in PBS-treated mice (Fig. 6e–g + Supplementary Fig. S6g,h; Table 1). At 8 weeks, hMAPC transplantation resulted in a significant 4-fold expansion of LYVE1^+^ lymphatic capillaries in the area surrounding the lymph node as compared to PBS-treatment (Fig. 6h–j). Finally, to test whether the beneficial effect of hMAPCs was related to functional reconnection of lymphatic collector vessels, we performed αSMA/Prox1 immunofluorescence stainings on cross-sections taken from the area around the transplanted lymph nodes and found lymph-filled Prox1^+^αSMA^+^ collectors (Fig. 6k–m). Collector identity was confirmed by negative staining for LYVE1 (Fig. 6n).

## Discussion

**Figure 7:**
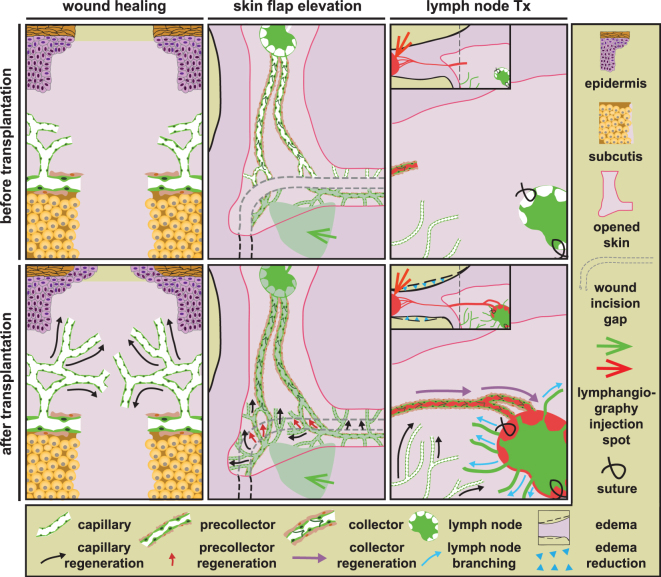
MAPCs support lymphatic regeneration at multiple anatomical levels. Following transplantation in different models, MAPCs stimulated lymphatic regeneration on three different anatomical levels. MAPCs stimulated capillary growth (black arrows) in all models and in addition boosted pre-collector regeneration in the skin flap model (red arrows) and collector restoration and lymph node (LN) reconnection (purple arrows) in the LN transplantation (Tx) model, thereby reducing edema (blue arrowheads). Finally, hMAPCs stimulated outward branching of the transplanted LN vasculature (blue arrows).

A cure for lymphedema is still lacking, despite its increasing prevalence among cancer survivors^43^. An optimal curative intervention requires the ability to regenerate the lymphatic system from the capillary to the (pre-)collector level^9^. However, current preclinical regenerative studies based on stem/progenitor cells are limited to models in which demonstration of functional restoration of lymphatic drainage is not necessarily dependent on repair of the lymphatic system at all anatomical levels. Furthermore, few - if any - studies have tested the lymphatic regenerative capacity of stem/progenitor cells in the context of wound healing. Here, we show that transplantation of MAPCs allows lymphatic regeneration at the capillary and (pre-)collector level and functionally reconnects lymph nodes to the host lymphatic network, suggesting that MAPCs could represent an attractive means to cure lymphedema (Fig. 7). In addition, MAPCs significantly improved the wound healing process in the epidermis and dermis likely in part by their combined stimulating effect on blood and lymphatic vessel growth in the wound granulation tissue.

We demonstrated that culturing MAPCs with VEGF-A results in a mixture of arterial, venous or lymphatic endothelial cells (this study and ref.^36^) and that exposure to VEGF-C or a combination of both did not increase lymphatic differentiation, unlike in mouse embryonic stem cells^44^ or human induced pluripotency stem cells^45^. This discrepancy may be related to differential expression of VEGFR3 – its expression being low in undifferentiated MAPCs. Likewise, their *in vivo* lymphatic endothelial differentiation and direct incorporation into lymphatic vessels was very infrequent, hence further studies are needed to identify optimal conditions for lymphatic endothelial cell differentiation of MAPCs. On the other hand, MAPC-conditioned media significantly boosted lymphatic endothelial cell proliferation, migration and sprouting *in vitro*. Accordingly, transplanted MAPCs, rather than being incorporated into lymphatic vessels, strategically positioned themselves – some persisting up to 6 months – near lymphatic vessels or transplanted lymph nodes from where they could deliver a complement of trophic factors supporting lymphatic regeneration. Indeed, MAPCs secreted a complex mixture of cytokines/growth factors, the composition of which was different between mMAPCs and hMAPCs. We hypothesise that the slightly better performance of hMAPC conditioned media *in vitro* during lymphatic endothelial cell proliferation and migration and the superior effect of hMAPCs in early functional restoration of lymph drainage *in vivo* in the skin flap elevation model (the only *in vivo* model in which we performed a side-by-side comparison of mMAPCs and hMAPCs) may be related to the more extensive cytokine/growth factor secretion profile - which included the key lymphangiogenic growth factor VEGF-C. Further studies are needed to identify the specific factors released by mMAPCs and hMAPCs responsible for their lymphangiogenic effect. We here confirmed our previous findings^37,38^ that mMAPCs and hMAPCs secrete significant levels of VEGF-A, shown to be responsible for the trophic effects of mesenchymal stem cells on lymphatic endothelial cells^46^, however, many other candidates may contribute in concert, including angiopoietins, hepatocyte growth factor, insulin growth factor binding proteins, (tissue inhibitors of) matrix metalloproteinases, stromal cell-derived factor-1, interleukin-8, osteopontin, and amphiregulin^47–51^.

To test the potential of MAPCs for lymphatic regeneration, we used an unprecedented combination of models with different stringencies to functionally restore the lymphatic system (Fig. 7). Other stem/progenitor cell-based studies have used non-physiological models (*e.g*., Matrigel plug implantation^31,52^) or models, *e.g*., tail circumcision or hind limb lymphatic cauterisation, in which the damage to the lymphatic system was less severe and the end-points of these studies generally were not related to stimulation of (pre-)collector growth or lymph node reintegration^22,30,34^. In two different models, we found that MAPCs stimulated lymphatic regeneration beyond the level of capillaries. While we showed that MAPCs support regeneration of the lymphatic tree at different anatomical levels (Fig. 7), it is currently unknown whether they supported (re)growth of (pre-)collectors directly or whether they mainly induced growth of capillaries which then subsequently matured into (pre-)collector vessels – a scenario that has been shown to occur after VEGF-C supplementation^9^. While the skin flap model developed by Saaristo *et al*. was considered as a model to test growth of lymphatic capillaries^9,42^, we found that the lymphangiogenic response to bridge the gap in the lymphatic system to some extent involved pre-collectors as well. In the second model, *i.e*., lymph node transplantation, hMAPCs supported restoration of lymphatic collectors. In our hands, we did not observe spontaneous lymph node drainage or regression of edema in PBS-treated mice, while others reported this upon transplantation in rodents^9,10,13,53^ or larger animal models^12,54^. This is most likely due to the fact that we used a more stringent model in which we only transplanted half lymph nodes devoid of a vascular pedicle and thoroughly eradicated the surrounding lymphatic network. The mechanisms by which hMAPCs supported functional lymph node reintegration were multi-faceted, including modes of action not previously documented in the context of spontaneous reintegration or VEGF-C adjuvant therapy. First, unlike VEGF-C supplementation^11^, hMAPC implantation increased the density of blood vessels surrounding the transplanted node, which may have contributed to its survival up to 6 months after transplantation^55^. Even though our *in vitro* studies provide evidence for a direct effect of MAPCs on lymphatic endothelial cell behaviour, the rather delayed effect on restoration of lymph node drainage may also imply that the effect of hMAPC transplantation on lymphatic regeneration was in part indirect through a prior angiogenic response. Secondly, combined lymph node and hMAPC transplantation not only increased the density of lymphatic vessels in the vicinity of the transplanted nodes but simultaneously induced remarkable and distant outward branching of the lymph node vasculature. This is different from other studies which have observed infiltration of surrounding donor vessels into the transplanted lymph node^53^ or formation of donor/recipient chimeric lymphatic vessels in close proximity to the transplanted nodes^9^.

In all models MAPCs had a combined angiogenic and lymphangiogenic effect, an appealing combination when aiming at improving wound healing^4^. Accordingly, hMAPCs significantly accelerated wound re-epithelialisation, granulation tissue formation and collagen remodelling. While lymphatic capillary growth following transfer of fibrin-binding VEGF-C did not improve re-epithelialisation, it was associated with increased granulation tissue formation and matrix remodelling in skin wounds independent of blood vessel growth^2^. It remains to be determined to what extent the latter two effects were co-determined by increased blood and lymphatic vessel growth following transfer of hMAPCs which supported growth of both vessel types. In the context of lymphedema caused by lymphatic ablation in cancer patients, such an additional proangiogenic effect may be unwanted as it could affect tumor growth or metastasis^10,56^. Furthermore, an effect on blood vessels could cause side effects, *e.g*. vascular leakage, as seen with high VEGF-C doses^57,58^, which may even promote lymphedema^58^. Hence, the proangiogenic response should be sufficiently balanced and locally controlled, rather than systemic. Importantly, we found that when mixed into Matrigel, hMAPCs could be strategically positioned around the lymph nodes thereby providing a local response. Moreover, upon local injection in the skin or muscle fascia, MAPCs stayed close to the injection spots. Furthermore, in all models the stimulating effect on lymphatic growth was slightly stronger than the effect on blood vessel growth (3-fold *versus* 2-fold).

In conclusion, MAPCs have a significant effect on lymphatic regeneration at all anatomical levels, which makes this progenitor cell type a strong candidate for treating lymphedema and stimulating combined blood vascular and lymphatic growth in healing wounds. Protocols to produce these cells in large scale and clinical grade format have been developed and the use of these cells has been shown to be safe in phase I/II trials^59^, which should accelerate the possibility to test MAPC treatment in lymphedema patients and in patients with chronic wounds.

## Methods

An extended methods section is provided in the supplement.

### Availability of materials and data

No datasets were generated or analysed during the current study.

### Cells

mMAPCs used in the current studies were derived from bone marrow of adult C57Bl/6 mice ubiquitously expressing eGFP, maintained under low O_2_/serum conditions and were previously characterised^37,60^. hMAPC populations were established and characterised at the University of Navarra (Pamplona; ‘hMAPC1’) and KU Leuven (‘hMAPC2’), as described^36,41^, after obtaining informed consent from the donors. Endothelial differentiation was performed by exposure to recombinant (r)hVEGF-A_165_ and/or rhVEGF-C, as described^36,41^. Procedures involving animals were approved by and performed in accordance with the guidelines of the Ethical Committee of the animal facilities at KU Leuven (approval number 005/2007 and 147/2010). Studies with hMAPCs complied with the Helsinki Declaration and were performed at KU Leuven after obtaining approval from and according to the guidelines of the Ethical Committee of University Hospitals Leuven (approval number B322201112107/S53482).

Human lung lymphatic endothelial cells (Lonza) were cultured in EBM2 supplemented with EGM-2-MV bullet kit. For conditioned media collection, MAPCs were seeded at high density in serum-free basal media and conditioned media was collected after 72 hours. Lymphatic endothelial cell proliferation, migration and sprouting assays were assessed in the presence of MAPC-conditioned or non-conditioned media, using a Ki67 staining (a list of antibodies is provided in Supplementary Table S2), modified Boyden chamber and collagen gel-based spheroid assays, respectively (see supplement).

### Antibody array, RNA isolation, cDNA preparation, qRT-PCR and flow cytometry

Antibody arrays were purchased from R&D Systems (ARY015 for mouse; ARY007 for human; cytokine/growth factors represented in the arrays are listed in Supplementary Table S1) and run according to the manufacturer’s instructions. Briefly, protein content in the 72 hour (non-)conditioned media was determined by BCA assay and equal amounts of protein were used for all conditions. Following overnight incubation, the signals of the retained proteins were revealed by a luminol-based detection reaction and quantified using Image Lab software. RNA was extracted using TRIzol. mRNA was reverse-transcribed using Superscript III Reverse Transcriptase and cDNA underwent 40 amplification rounds (primer sequences are listed in Supplementary Table S3) on an ABI PRISM7700 cycler. For LYVE1 protein expression, cells were analysed by FACS (see supplement).

### Mouse models

As MAPCs do not express Major Histocompatibility Complex-I (MHC-I) and are sensitive to natural killer cell-mediated clearance, all mice were injected intraperitoneally with anti-asialo GM1 antibodies 1–2 hours before transplantation and every 10 days thereafter to selectively eliminate natural killer cells.

#### Linear wound model

At day 0, a 12-mm linear skin incision was inflicted on the back of anaesthetised 12 week-old C57Bl/6 males and mice were injected locally with 1 × 10^6^ mMAPCs or PBS. Wound dimensions were measured daily using digital calipers and pictures were taken. At day 4, bright field and fluorescence pictures of the wound area were taken. At day 10, mice were euthanised and the residual skin wound and underlying muscle tissue were prepared for embedding.

#### Circular wound model

At day 0, 12 week-old athymic nude Foxn1 males were anaesthetised and standardised full-thickness wounds were made with a biopsy puncher (Stiefel Laboratories) on their back. A silicone ring was sutured around the wound and wounds were treated with PBS or 5 × 10^5^ hMAPCs. In a subset of mice, hMAPCs were transduced with an eGFP-encoding lentivirus before transplantation. An occlusive Tegaderm dressing was used to keep the wound moist and was renewed every other day and pictures were taken. Wound size was measured using ImageJ software and was expressed as the % *versus* the size at day 0 for each individual mouse. At 5 or 10 days after wounding, mice were euthanised, skin wounds were dissected out and processed for embedding.

#### Skin flap model

At day 0, 12 week-old athymic nude Foxn1 males were anaesthetised and the abdominal skin lymphatic network was severed by elevating an epigastric skin flap and suturing it back to its original position^42^. One day after resuturing, 1 × 10^6^ MAPCs or PBS were injected around the wound edges. Two or 4 weeks later, axillary regions were exposed and lymph node drainage was monitored by microlymphangiography after intradermal injection of FITC-dextran (hMAPCs) or Rhodamin-B-isothiocyanate-dextran (mMAPCs) under the wound border. Bright field and fluorescence pictures were taken at 15 minutes and mice were subsequently euthanised, the skin wound area around the cell engraftment/microlymphangiography areas excised and processed for embedding.

#### Lymph node transplantation model

At day 0, 12 week-old athymic nude Foxn1 females were anaesthetised, the right axilla regions exposed and lymph nodes removed (along with the surrounding lymphatic (collector) vessels). Donor lymph nodes were dissected from mice ubiquitously expressing DsRed (for mice receiving hMAPCs or PBS and followed up for 4 or 8 weeks) or eGFP (for mice receiving hMAPCs or PBS and followed up for 4 or 16 weeks), cut in two halves through the hilus, one half was implanted into a pocket just caudal to the right axillary vessels and fixed in place with sutures. Cold growth factor-reduced Matrigel mixed with 0.5 × 10^6^ hMAPCs or PBS was applied into the pocket and allowed to solidify. The skin was closed and the wound covered with Tegaderm dressing. Four, eight or sixteen weeks later, mice were anaesthetised and subjected to microlymphangiography following injection of FITC-conjugated or Texas Red-conjugated *L. esculentum* lectin (in DsRed^+^ or eGFP^+^ lymph node recipients, respectively). Implanted lymph node drainage was monitored for 15 minutes and bright field and fluorescence pictures were taken at the end. Mice were subsequently euthanised, axilla regions containing the transplanted lymph node excised and processed for embedding. Additional sets of mice were subjected to *in vivo* magnetic resonance imaging (MRI; see supplement).

### Histology and morphometry

Morphometry, immunofluorescence and immunohistochemistry staining procedures are described in the supplement and a list of primary antibodies is provided in Supplementary Table S2.

### Statistics

Quantitative data represent mean ± s.e.m. ‘N’ represents the number of independent biological replicates on which statistical tests were performed. For qRT-PCR, measurements were performed in technical duplicate and averaged for each biological replicate. Tests used for statistical analyses are mentioned in the results and figure legends text. Data normality was tested by the Shapiro-Wilk test. Comparisons among two groups were performed by unpaired two-tailed Student’s *t*-test in case of normal distribution or by Mann-Whitney-U test in cases where data were not normally distributed or normality could not be assumed. Multiple-group comparisons were done by 1-way ANOVA with Tuckey’s post-hoc test (normal distribution) or Kruskal-Wallis test followed by Dunn’s post-hoc test (no normality assumption). Wound size, width or length were evaluated by repeated measures ANOVA, followed by Fisher least-significant-difference test. Data were considered significant if the *P*-value was less than 0.05. All analyses were performed with Graphpad Prism (version 6.0).

## Electronic supplementary material

Supplementary information
