# Supplementary material for: Multipotent Adult Progenitor Cells Support Lymphatic Regeneration at Multiple Anatomical Levels during Wound Healing and Lymphedema
Source: Sci Rep. 2018 Mar 1;8:3852. doi: 10.1038/s41598-018-21610-8 (PMC5832783; doi:10.1038/s41598-018-21610-8)
Supplement: Supplementary file 1 — Supplementary information [file 41598_2018_21610_MOESM1_ESM.pdf]

SUPPLEMENT
to

Multipotent Adult Progenitor Cells Support Lymphatic Regeneration at Multiple Anatomical Levels during Wound Healing and Lymphedema

Authors:

Manu Beerens, Xabier L. Aranguren, Benoit Hendrickx, Wouter Dheedene, Tom Dresselaers, Uwe Himmelreich, Catherine Verfaillie, Aernout Luttun

A. EXTENDED METHODS

Cells

MAPC derivation and differentiation. Mouse (m)MAPCs were derived from bone marrow of adult C57Bl/6 mice with ubiquitous eGFP expression (C57Bl/6-Tg-eGFP; a gift by Dr. I. Weissman, Stanford, USA). mMAPCs were derived and maintained under low O_2 (5%) and low-serum (2%) conditions and characterised, as previously described[1,2]. Human (h)MAPC populations were established and characterised at the University of Navarra (Pamplona; 'hMAPC1') or at the Stem Cell Institute (KU Leuven; 'hMAPC2'), as described earlier[3,4], after obtaining informed consent from the donors. Cell cultures were routinely tested for mycoplasma contamination. Endothelial differentiation was performed by exposure of the cells to recombinant (r)hVEGF-A_{165} and/or rhVEGF-C (both from R&DSystems), as described[4]. Procedures involving animals were approved by and performed in accordance with the guidelines of the Ethical Committee of the animal facilities at KU Leuven (approval number 005/2007 and 147/2010). Studies with hMAPCs complied with the Helsinki Declaration and were performed at KU Leuven after obtaining approval from and according to the guidelines of the Ethical Committee of University Hospitals Leuven (approval number B322201112107/S53482).

Lymphatic endothelial cell culture and conditioned media collection. Human lung lymphatic endothelial cells were purchased from Lonza (Merelbeke, Belgium) and cultured in EBM2 supplemented with EGM-2-MV bullet kit (Lonza). For conditioned media collection, MAPCs were seeded at high density in serum-free basal media and conditioned media was collected after 72 hours and frozen in aliquots at -80°C until further use.

Lymphatic endothelial cell proliferation. To test the effect of MAPC-conditioned media on lymphatic endothelial cell proliferation, lymphatic endothelial cells were seeded at a density of 4,000 cells/cm^2 in regular lymphatic endothelial cell growth medium onto gelatin-coated 96-well plates. On day 2, medium was replaced with serum-free lymphatic endothelial cell media for 4 hours. After washing with PBS, cells were incubated with either MAPC-conditioned media or non-conditioned

media as reference condition, both supplemented with 1% FBS. After 48 hours, cells were fixed with 100% methanol, permeabilised with Triton-X-100 and stained overnight with anti-Ki67 primary antibody (Supplementary Table S2). The day after, cells were washed, incubated with Alexa488-conjugated secondary antibody and Hoechst for nuclear staining. Proliferation index was determined by counting the fraction of Ki67$^+$ cells of the total (Hoechst$^+$) cells in 5 randomly chosen fields taken per replicate with a Zeiss MRm camera mounted onto an Axiovert200M microscope and equipped with Axiovision 4.8 software, at 20x magnification (using an LD Plan-Neofluar objective lens, NA 0.4) by a blinded observer.

Lymphatic endothelial cell migration. To estimate the effect of MAPC-conditioned media on lymphatic endothelial cell migration, a Boyden chamber assay was performed. Briefly, transwell inserts (containing polycarbonate filters with 8 µm pore size; Costar, Corning) were coated overnight with 0.2% gelatin. The bottom compartment of a 24-well plate was filled with 0.3 ml non-conditioned media or with 0.3 ml of mMAPC- or hMAPC-conditioned media. Following rehydration for 1 hour with deionised water, inserts were placed into the 24-well plate and each was loaded with 0.3 ml EGM-2-MV/0.5% FBS containing 5x10^4 lymphatic endothelial cells. Following incubation for 24 hours at 37°C/5% CO_2, cells were fixed in methanol for 30 minutes at -20°C. Next, cells were stained with Wright-Giemsa's staining solution (Sigma WG32) for 7 minutes and rinsed with deionised water for 10 minutes. Inserts were lifted and cells on the upper side of the membranes were removed by gentle rubbing using a cotton swab. Pictures of the inserts were taken with a Zeiss MRc5 camera mounted onto an Axiovert200M microscope and equipped with Axiovision 4.8 software, and transmigrated cells were manually counted in 3 random fields per insert at 20x magnification (using an LD Plan-Neofluar objective lens, NA 0.4) by a blinded observer.

Lymphatic endothelial cell sprouting. To test the effect of mMAPC-conditioned media on lymphatic endothelial cell sprouting, lymphatic endothelial cell spheroids were allowed to form by applying 25 µl droplets (containing 1,000 lymphatic endothelial cells in a 20% methylcellulose/EGM-2-MV mixture) onto non-attachment plates and incubating them upside down at 37°C/5%CO_2. The

next day, spheroids were carefully washed in PBS/2%FBS, collected by gentle centrifugation, carefully resuspended in methylcellulose/FBS/collagen (Purecol Advanced Biomatrix) and seeded into 24-well plates (0.5 ml/well). Following incubation of 30 minutes at 37°C/5% CO_2, 0.5 ml mMAPC-conditioned media (1:1 mix with serum-free lymphatic endothelial cell media) or 100% serum-free lymphatic endothelial cell media as reference condition was added on top of the collagen/spheroid gel. Pictures were taken 24 hours later at 20x magnification (using an LD Plan-Neofluar objective lens, NA 0.4) with a Zeiss MRm camera mounted on a Zeiss Axiovert200M microscope and the number of sprouts per spheroid was determined by manual counting by a blinded observer.

Antibody array, RNA isolation, cDNA preparation, quantitative (q)RT-PCR and flow cytometry

Antibody arrays were purchased from R&D Systems (ARY015 for mouse; ARY007 for human; cytokine/growth factors represented in the arrays are listed in Supplementary Table S1 online) and run according to the manufacturer's instructions. Briefly, protein content in the 72 hour (non-)conditioned media was determined by BCA assay and equal amounts of protein were used for all conditions. Following overnight incubation, the signals of the retained proteins were revealed by a luminol-based detection reaction using a ChemiDoc XRS+ molecular imager (Bio-Rad) and quantified using Image Lab software (version 4.0, Bio-Rad Labs). For quantification, arrays of the conditioned media (n=3) and the corresponding non-conditioned media (n=1) were developed together and mean pixel density in every spot of the array was determined. The Δ mean pixel density was calculated by subtracting the value of the non-conditioned media from the corresponding values of the conditioned media, averaged and expressed in arbitrary units. To determine % overlap between species, we determined for those proteins that were common analytes on both arrays whether or not there was a signal detected above that of the non-conditioned media.

Total RNA from cell lysates was extracted using TRIzol reagent (Invitrogen). mRNA was reverse transcribed using Superscript III Reverse Transcriptase (Invitrogen) and cDNA underwent 40

amplification rounds on an ABI PRISM7700 cycler PerkinElmer/Applied Biosystems) for SYBR-Green-based qRT-PCR, as described[5]. Primer sequences for qRT-PCR are listed in Supplementary Table S3. mRNA levels were normalised using *GAPDH* as house-keeping gene. To analyse LYVE1 expression on the surface of differentiated mMAPCs, cells were harvested by gentle trypsinisation, washed with FACS staining buffer (PBS + 1 mmol/L EDTA + 25 mmol/L Hepes + 1% BSA) and incubated with primary antibody (Upstate, 07-538) or the corresponding rabbit IgG isotype for 20 minutes at room temperature in the dark. After washing with FACS buffer, cells were incubated with biotinylated goat-anti-rabbit secondary antibodies for 20 minutes at room temperature in the dark. Next, samples were washed and incubated in the dark for 20 minutes with allophycocyanin (APC)-labelled streptavidin. To select for viable cells, 7-AAD was added 10 minutes before running the samples on a FACS Aria I (Beckton Dickinson) for analysis.

Mouse models

As MAPCs do not express Major Histocompatibility Complex-I and – consequently – are sensitive to natural killer cell-mediated clearance, all mice (including PBS controls) were injected intraperitoneally with 20 μl anti-asialo GM1 antibodies (Wako Chemicals, Osaka, Japan; 20x diluted in PBS) 1-2 hours before transplantation and every 10 days thereafter. These antibodies selectively eliminate natural killer cells without affecting macrophage or lymphocyte function[6].

Linear wound model: At day 0, a 12-mm linear skin incision was inflicted with a scalpel on the back of 12 week-old C57Bl/6 male mice after they were anaesthetised with a mixture of 100 mg/kg ketamine and 10 mg/kg xylazine. Immediately after wounding, mice were injected in the muscle fascia underneath the skin wound with $1x10^6$ mMAPCs (resuspended in PBS) or PBS alone divided over three equally spaced injection spots. To avoid wound infection, mice were housed individually in cages without bedding. Wound dimensions were measured daily under isoflurane anaesthesia using digital calipers (VWRI819-0012, VWR) and pictures were taken using a NikonD1 camera and Camera-Control-Pro software. At day 4, bright field and fluorescence pictures of the wound area were taken with a Zeiss MRc5 camera mounted on a Zeiss Lumar microscope (using a

Neolumar S lens, 0.8x magnification, FWD 80 mm). At day 10, mice were euthanised, the residual skin wound and underlying muscle tissue were dissected out, fixed in zinc-paraformaldehyde and prepared for embedding in paraffin or optimal cutting temperature (OCT) and sectioning.

Circular wound model: At day 0, 12 week-old athymic nude Foxn1 male mice (Harlan) were anaesthetised with an intraperitoneal injection of ketamine (100 mg/kg) and xylazine (10 mg/kg). Atropine (0.01 mg/kg) was administered intraperitoneally as premedication. Under sterile and temperature-controlled (37°C) conditions, standardised full-thickness wounds were made with a 0.5 cm biopsy puncher (Stiefel Laboratories, Offenbach am Main, Germany) on the back of the mouse in the mid-dorsal region. A silicone ring was fixed (using Histoacryl tissue adhesive, Braun, Diegem, Belgium) and sutured around the wound and wounds were treated with PBS or $5x10^5$ hMAPCs. In a separate subset of mice, hMAPCs were transduced with an eGFP-coding lentivirus prior to transplantation. An occlusive dressing (Tegaderm, 3M, Diegem, Belgium) was used to keep the wound moist. All wounded mice were housed individually to avoid fighting and to prevent removal of the occlusive wound dressing. Every other day, the occlusive dressing was renewed under isoflurane anaesthesia and pictures were taken using a NikonD1 camera and Camera-Control-Pro software. Wound size was measured using Image J software and was expressed as the *% versus* the size at day 0 for each individual mouse. At 5 or 10 days after wounding, mice were euthanised and square skin fragments including the circular wound area and a rim of normal skin were dissected out, rinsed in PBS and post-fixed overnight at 4°C using zinc-paraformaldehyde. Following fixation, skin fragments were separated in two equal pieces at the midline of the wound and processed for paraffin or OCT embedding and sectioning.

Skin flap model: At day 0, 12 week-old athymic nude Foxn1 male mice (Harlan) were anaesthetised with an intraperitoneal injection of ketamine (100 mg/kg) and xylazine (10 mg/kg). The lymphatic network in the abdominal skin was severed by elevating an epigastric skin flap and suturing it back to its original position, as previously described[7]. Continuous blood supply to the flap was ensured by retaining a vascular pedicle including the right inferior epigastric artery and vein (Fig.

3a). One day after resuturing the skin flap, 1x10^6 mMAPCs, 1x10^6 hMAPCs or PBS (divided over 4 injection spots; Fig. 3a) were injected around the wound edges. Two or 4 weeks later, the axillary regions were exposed and axillary lymph node drainage was monitored by microlymphangiography for 15 minutes after intradermal injection of 10 µl FITC-dextran (mol wt 2,000 kDA, Sigma-Aldrich; hMAPCs) or 10 µl Rhodamin-B-isothiocyanate-dextran (mol wt 70 kDA, Sigma-Aldrich; mMAPCs) under the wound border (Fig. 3a). Bright field and fluorescence pictures were taken at 15 minutes with a Zeiss MRc5 camera mounted onto a Zeiss Lumar microscope (using a Neolumar S lens, 0.8x magnification, FWD 80 mm). Mice were subsequently euthanised, the skin wound area around the cell engraftment/microlymphangiography areas excised, fixed and processed for paraffin or OCT embedding and sectioning.

Lymph node transplantation model: At day 0, 12 week-old athymic nude Foxn1 female recipient mice (Harlan) were anaesthetised with an intraperitoneal injection of ketamine (100 mg/kg) and xylazine (10 mg/kg). To visualise the lymph nodes, the right axilla region was exposed and mice were injected with a 3% Evans Blue solution in the palm of the right paw after which lymph nodes were removed along with the surrounding lymphatic (collector) vessels. A pocket just caudal of the axillary vessels, aligned by the lateral axillary fat pad, the *M. pectoralis* and the *M. latissimus dorsi* was prepared. Donor lymph nodes were dissected from mice ubiquitously expressing DsRed (B6.Cg-Tg(CAG-DsRed*MST)1Nagy/J; for mice receiving hMAPCs or PBS and followed up for 4 or 8 weeks) or eGFP (C57Bl/6-Tg(CAG-EGFP)1Osb/J; for mice receiving hMAPCs or PBS and followed up for 4 or 16 weeks) and cut in two halves through the hilus. The cut lymph node was subsequently implanted into the recipient pocket (hilus oriented medially and cut surface facing upwards) and fixed in place with two permanent sutures (using 9-0 nylon non-absorbable suture, Monosof). Cold growth factor-reduced Matrigel (100 µl; Beckton Dickinson) mixed with 0.5x10^6 hMAPCs or PBS was applied into the pocket and allowed to solidify for 10 minutes. The skin was subsequently closed and the wound covered with Tegaderm dressing. Four, 8 or 16 weeks later, mice were anaesthetised with a ketamine/xylazine mixture and subjected to microlymphangiography following injection of 10

µl FITC-conjugated *L. esculentum* lectin (Vector Laboratories; in recipients of $DsRed^+$ donor lymph nodes) or 10 µl Texas Red-conjugated *L. esculentum* lectin (in recipients of $eGFP^+$ lymph nodes) in the palm of the right paw. Drainage of the implanted lymph node was monitored for 15 minutes and bright field and fluorescence pictures were taken at the end with a Zeiss MRc5 camera mounted onto a Zeiss Lumar microscope (using a Neolumar S lens, 0.8x magnification, FWD 80 mm). Mice were subsequently euthanised, the axilla regions containing the transplanted lymph node excised, fixed and processed for paraffin or OCT embedding and sectioning. Two additional sets of mice were subjected to *in vivo* magnetic resonance imaging (MRI), as described[8] at 4 or 16 weeks after lymph node transplantation. Briefly, mice were anaesthetised with isoflurane and mustard oil (diluted 1/5 in mineral oil) was applied with a cotton stick on both fore limbs for 2 x 15 minutes to elicit vascular hyperpermeability and aggravate edema. Mice were allowed to recover for another 30 minutes before MRI recording. Temperature and respiration were monitored throughout the experiment and maintained at 37°C and 100 - 120 breaths per minute. MR images were acquired with a 9.4 T Biospec small animal MR scanner (Bruker Biospin, Ettlingen, Germany) equipped with a horizontal bore magnet and an actively shielded gradient set of 600 mT per m (117 mm inner diameter) using a 7 cm linearly polarised resonator for transmission and an actively decoupled dedicated 2 cm diameter surface coil for receiving (Rapid Biomedical, Rimpar, Germany). After the acquisition of 2D localisation scans; 3D T_2 weighted images, 2D T_2 parameter maps and 2D diffusion weighted images were acquired to determine the level of edema. Specific parameters were: 3D rapid acquisition with refocused echoes (RARE) sequence, repetition time (TR): 1,300 ms, effective echo time (TE): 22.9 ms, rare factor: 6, matrix size: 256x48x48, field of view (FOV): 2.5x0.7x1.5 cm, resolution: 98x146x312 μm^3; 2D T_2 maps: TR: 3,500 ms, 10 TE's between: 10 - 100 ms, matrix size: 256x256, FOV: 2x2 cm, 15 transverse slices with slice thickness: 0.3 mm and gap 0.3 mm, in plane resolution: 78 μm^2; diffusion weighted MRI: spin echo sequence; b-value of 1,500 s mm^{-2}, TR: 25 ms, TE: 3,000 ms, matrix size: 128x128, FOV: 2x2 cm, 8 transverse slices of 1 mm thickness. Processing of the 3D T_2 weighted images was done by determining the volume with a signal intensity above a common

threshold value using home-written software developed with Mevislab (Mevis Medical Solutions, Bremen, Germany) reported as ratios between the lymph node implanted side *versus* the control side. Calculation of the T_2 parameter maps of the manually delineated edema of the paws (or an area of the same size and located in the same region in the absence of edema) was done using Paravison 5.1 (Bruker Biospin).

Histology and morphometry

Morphometric analyses were performed on 7 µm paraffin sections, 10 µm cryosections or bright field pictures of exposed skin regions by blinded observers. Lymphatic (determined on LYVE1-, podoplanin-, Flt4- or Prox1/αSMA-stained sections) or blood (determined on CD31-stained sections) vessel density, collagen content/organisation (determined on Sirius red-stained sections) and epithelial coverage and granulation tissue formation (both determined on pancytokeratin (PCK)-stained sections) was scored on at least 10 randomly chosen fields per mouse, covering a distance of 700 µm. Functional lymphatics (determined on cryosections of mice injected with fluorescently-labelled dextran) were counted on 8-10 consecutive sections per mouse, thereby scanning the entire wound area visible on each section. The fractional area of the blood vessel network leading up to the transplanted lymph nodes was determined on digitally reconstructed images of the entire region of interest. For stainings on paraffin sections, slides were deparaffinated and rehydrated, cryosections were incubated in PBS for five minutes prior to the staining procedure. Haematoxylin&Eosin and Sirius red staining were performed as previously described[1]. For CD31, podoplanin, Flt4 or PCK immunohistochemical staining, antigen retrieval was performed by boiling in target retrieval solution s1699 (Sigma). After cooling down in TBS, endogenous peroxidase activity was quenched in 0.3% H_2O_2 in methanol. Slides were incubated with primary antibody overnight. A list of primary Ab's is provided in Supplementary Table S2. After washing in TBS, slides were incubated for 2 hours with biotinylated rabbit-anti-rat (CD31 and Flt4), goat anti-mouse (PCK) or goat-anti-hamster (podoplanin) antibodies and the detection signal was amplified with a tyramide signal amplification system (Perkin Elmer, NEL700A). Nuclei were revealed by

haematoxylin counterstaining and slides were mounted with DPX mountant (Sigma). For LYVE1 immunofluorescence staining, antigen retrieval was performed by boiling in target retrieval solution s1699 (Sigma). After cooling down in TBS, endogenous peroxidase activity was quenched in 0.3% H_2O_2 in methanol. Slides were incubated with primary antibody overnight. After washing in TBS, slides were incubated for 2 hours with biotinylated goat-anti-rabbit antibody and the detection signal was amplified with a tyramide-Cy3 or tyramide-fluorescein signal amplification system (Perkin Elmer, NEL704A or NEL701A). When combined with CD45 immunofluorescence staining, slides were subsequently incubated with primary anti-CD45 antibody overnight, followed by a 2 hour incubation with goat-anti-rat-Alexa488. For eGFP or vimentin immunofluorescence staining, antigen retrieval was performed by boiling in citrate buffer (pH=6). After overnight incubation with primary antibody, slides were incubated for 1 hour with Alexa-conjugated donkey-anti-chicken (eGFP) or goat-anti-mouse (vimentin) antibodies. For combined LYVE1/vimentin immunofluorescence staining, antigen retrieval was performed by boiling in citrate buffer (pH=6) and tissues were permeabilised by incubation in Triton 0.1% in PBS. After overnight incubation with primary antibodies, slides were incubated for 1 hour with goat-anti-mouse-Alexa488 and goat-anti-rabbit-Alexa568. For combined Prox1/αSMA immunofluorescence staining, antigen retrieval was performed by boiling in citrate buffer (pH=6) and tissues were permeabilised by incubation in Triton 0.1% in PBS. After overnight incubation with Prox1 primary antibody, slides were incubated for 1 hour with biotin-conjugated goat-anti-rabbit Ab and the detection signal was amplified with a tyramide-Cy3 or tyramide-fluorescein signal amplification system (Perkin Elmer, NEL704A or NEL701A). Slides were subsequently stained with Cy3-conjugated αSMA for 2 hours or with unconjugated SMA followed by goat-anti-mouse-Alexa660. For combined Prox1/eGFP immunofluorescence staining, antigen retrieval was performed by boiling in citrate buffer (pH=6) and tissues were permeabilised by incubation in Triton 0.1% in PBS. After overnight incubation with Prox1 and eGFP primary antibodies, slides were incubated for 1 hour with biotin-conjugated goat-anti-rabbit and Alexa488-conjugated donkey-anti-chicken antibodies and the Prox1 detection signal

was amplified with a tyramide-Cy3 signal amplification system (Perkin Elmer). Combined mCD31 and hCD31 staining was performed as previously described[9]. Immunofluorescence-stained slides were sealed with ProLong Gold Antifade Reagent with DAPI (Life Technologies; P36931). Images were recorded on a Zeiss Axiovert 200M microscope (at 20x or 40x magnification with LD Plan-NeoFluar lenses with NA 0.4 and NA 0.6, respectively), a Zeiss Axio Imager Z1 microscope equipped with a Zeiss MRc5 camera (at 10x, 20x or 40x magnification with EC Plan-NeoFluar lenses with NA 0.3, NA 0.5 and NA 0.75, respectively) or a Leica Leitz DMRBE equipped with a Zeiss MRc5 camera (at 5x or 20x magnification with a N Plan lens, NA 0.11 and a PL Fluotar lens, NA 0.5, respectively) and Axiovision 4.8 software. Images were cropped, pseudo-coloured and contrast adjusted using Photoshop (Adobe).

Statistics

Quantitative data represent mean ± s.e.m. 'N' represents the number of independent biological replicates on which statistical tests were performed. For qRT-PCR, measurements were performed in technical duplicate and averaged for each biological replicate. Tests used for statistical analyses are mentioned in the results and figure legends text. Normality of the data was tested by the Shapiro-Wilk test. Comparisons among two groups were performed by unpaired two-tailed Student's *t*-test in case of normal distribution or by Mann-Whitney-U test in cases where data were not normally distributed or normality could not be assumed. Multiple-group comparisons were done by 1-way ANOVA with Tuckey's post-hoc test (normal distribution) or Kruskal-Wallis test followed by Dunn's post-hoc test (no normality assumption). Wound size, length and width were evaluated by repeated measures ANOVA, followed by Fisher least-significant-difference test. Data were considered significant if the *P*-value was less than 0.05. All analyses were performed with Graphpad Prism (version 6.0).

B. SUPPLEMENTARY FIGURES

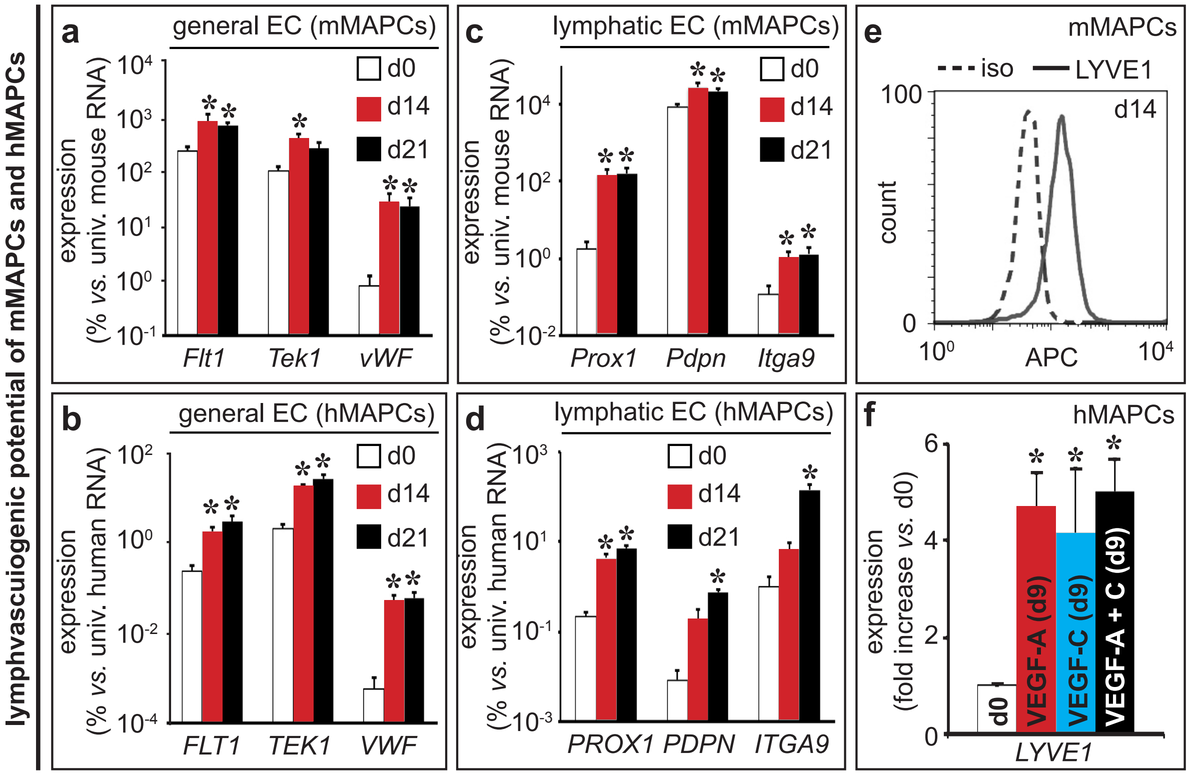

Supplementary Figure S1. MAPCs differentiate down the lymphatic endothelial lineage. a-d, Expression of general endothelial cell (EC) markers (*a,b*) or lymphatic-EC-specific markers (*c,d*), shown as % *versus* universal mouse RNA in undifferentiated (day (d)0, white) mMAPCs (*a,c*) or universal human RNA in undifferentiated (d0, white) hMAPCs (*b,d*), at 14 (d14, red) and 21 (d21, black) days of differentiation; *n*=4-7 independent differentiations. *P*=0.023 for *Flt1*, *P*=0.0094 for *Tek1*, *P*=0.028 for *vWF*, *P*=0.0039 for *FLT1*, *P*=0.0018 for *TEK1*, *P*=0.0022 for *VWF*, *P*=0.0016 for *Prox1*, *P*=0.0013 for *Pdpn*, *P*=0.014 for *Itga9*, *P*<0.0001 for *PROX1*, *P*=0.0002 for *PDPN*, *P*=0.0012 for *ITGA9* by Kruskal-Wallis test; **P*<0.05 *versus* d0 by Dunn's post-hoc test. **e,** FACS histogram (representative of *n*=3 independent differentiations) showing LYVE1 expression (full line) *versus* isotype control (dashed line) in mMAPCs at d14. APC: allophycocyanine. **f,** *LYVE1* expression, shown as fold-increase *versus* undifferentiated hMAPCs (d0, white), or at d9 in the presence of VEGF-A (red), VEGF-C (blue) or a combination (black); *n*=3 independent differentiations; *P*=0.0012 by 1-way ANOVA; **P*<0.05 *versus* d0 by Tuckey's post-hoc test.

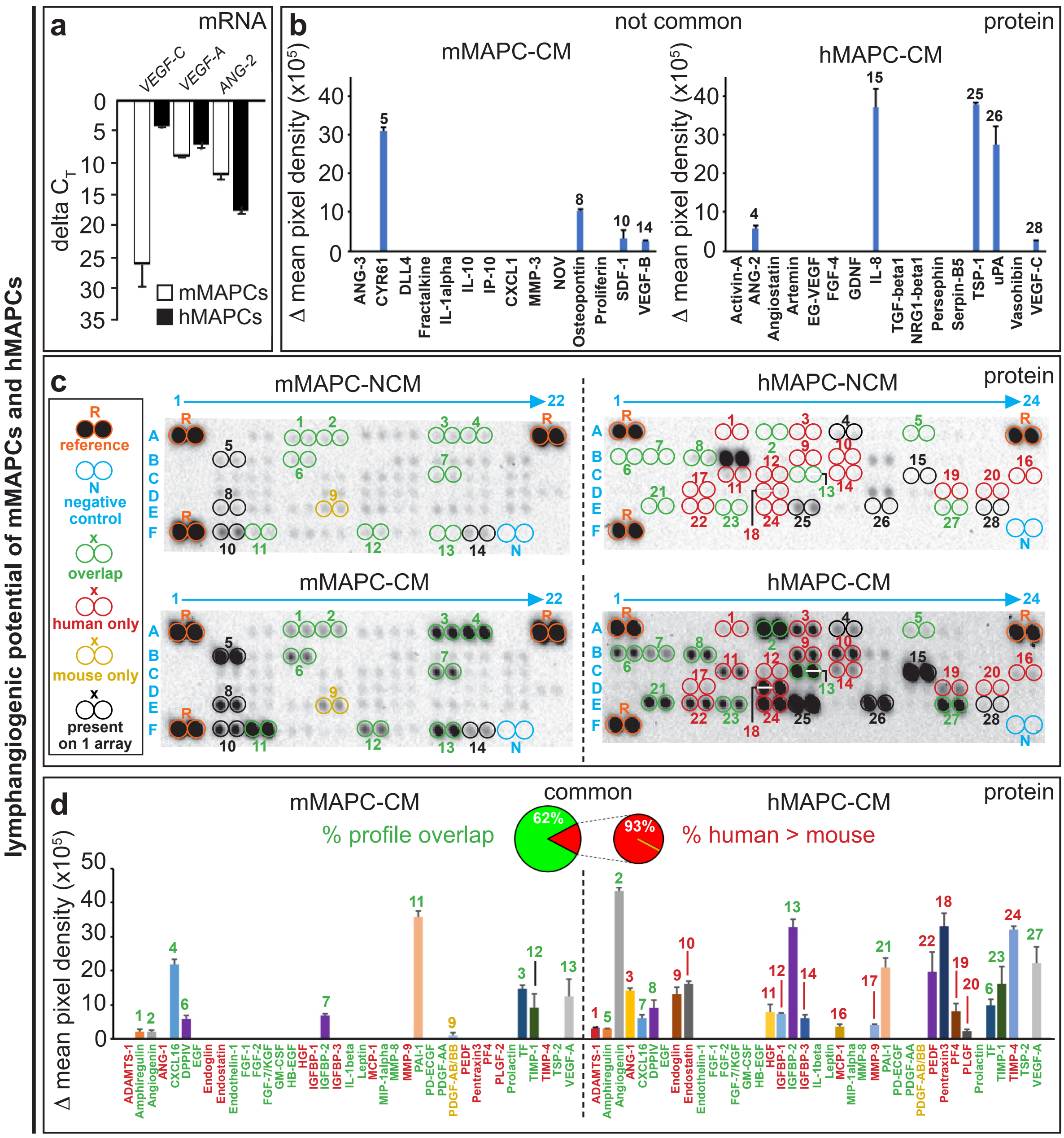

Supplementary Figure S2. mMAPCs and hMAPCs have complex, partially overlapping secretomes. a, Diagram showing mRNA expression for lymphangiogenic growth factors in mMAPCs (white bars) and hMAPCs (black bars), determined by qRT-PCR and expresssed as delta C_T; *n*=3. **b-d,** Representative antibody arrays on conditioned media (CM; *c*, bottom) or non-conditioned media (NCM; *c*, top) for mMAPCs (*c*, left) and hMAPCs (*c*, right) and corresponding quantification of proteins not commonly (*b*) or commonly (*d*) represented on mouse and human arrays (expressed as Δ mean pixel density (= mean of CM signal - NCM signal); *n*=3; note: negative values are not shown). Numbered and encircled spots on the arrays correspond to proteins that were detected above corresponding NCM level (proteins commonly secreted by mMAPCs and hMAPCs are in green, proteins only secreted by hMAPCs are in red, proteins only secreted by mMAPCs are in gold, secreted proteins that were only represented on 1 of the array types are in black; reference spots are

in orange; negative control spots are in blue). The left pie-diagram in *d* represents the overlap (in terms of being secreted or not; in green) between the two species. The right pie-diagram in *d* shows that in case of non-overlap, the protein was secreted only by hMAPCs in 93% of the cases (in red) and in 7% of the cases only by mMAPCs (in gold). The fully annotated list of proteins and corresponding coordinates on the arrays are shown in Supplementary Table S1.

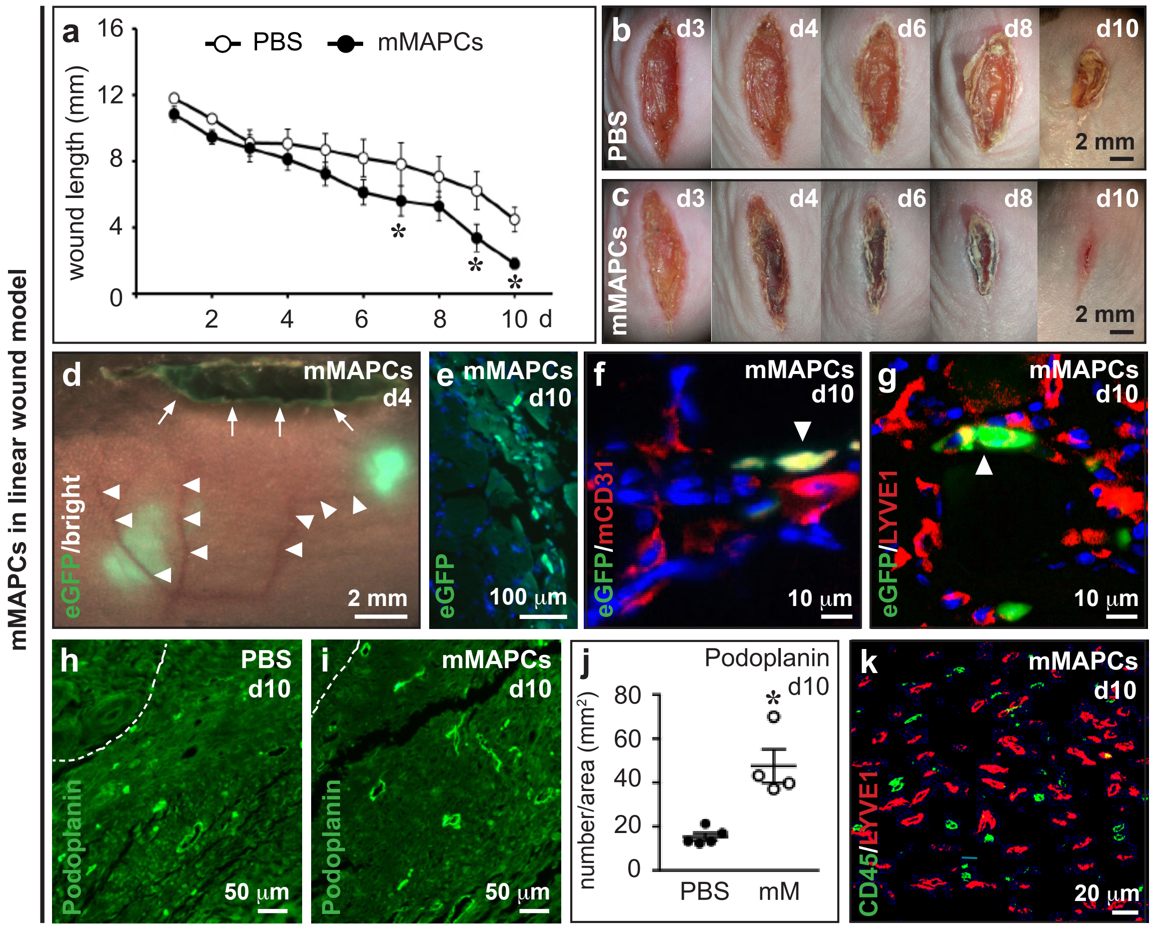

Supplementary Figure S3. mMAPCs promote wound healing. a-c, Diagram (*a*) representing wound length (in mm) in mice treated with PBS (n=5: open circles) or mouse (m)MAPCs (n=5; filled circles) until 10 days (d) after wounding and representative bright field pictures of the wounds on d3, d4, d6, d8 and d10 after wounding of a PBS-treated (*b*) or mMAPC-treated (*c*) mouse. Quantitative data represent mean ± s.e.m. *P<0.05 *versus* PBS by repeated measures ANOVA with Fisher post-hoc test. **d,** Merged bright field/fluorescence image of the back skin of a mouse transplanted 4 d earlier with eGFP$^+$ mMAPCs. Arrows indicate the wound border, arrowheads indicate blood vessels leading up to the wound area. Large green fluorescent spots correspond to clusters of transplanted eGFP$^+$ cells locating in the vicinity of blood vessels. **e,** Cross-section of the area underneath the wound showing individual eGFP$^+$ mMAPCs at 10 d after transplantation. **f,g,** Pictures of cross-sections of 10 d-old wounds from mice treated with eGFP$^+$ mMAPCs (in green) co-stained with anti-mouse (m)CD31 (in red; *f*) or LYVE1 (in red; *g*). White arrowheads indicate co-localisation (in yellow). **h-j,** Podoplanin-stained (in green) cross-sections of 10 d-old wounds treated with PBS (*h*) or mMAPCs (*i*), and corresponding quantification (*j*; expressed as number of vessels per area in mm^2; n=4-5; *P=0.0024 *versus* PBS by unpaired two-tailed Student's t-test). **k,** Merged picture of a red and green fluorescence image of a wound cross-section revealing no co-localisation of CD45 (in green) with LYVE1 (in red). TOPRO3 was used as nuclear counterstain in *e-g*.

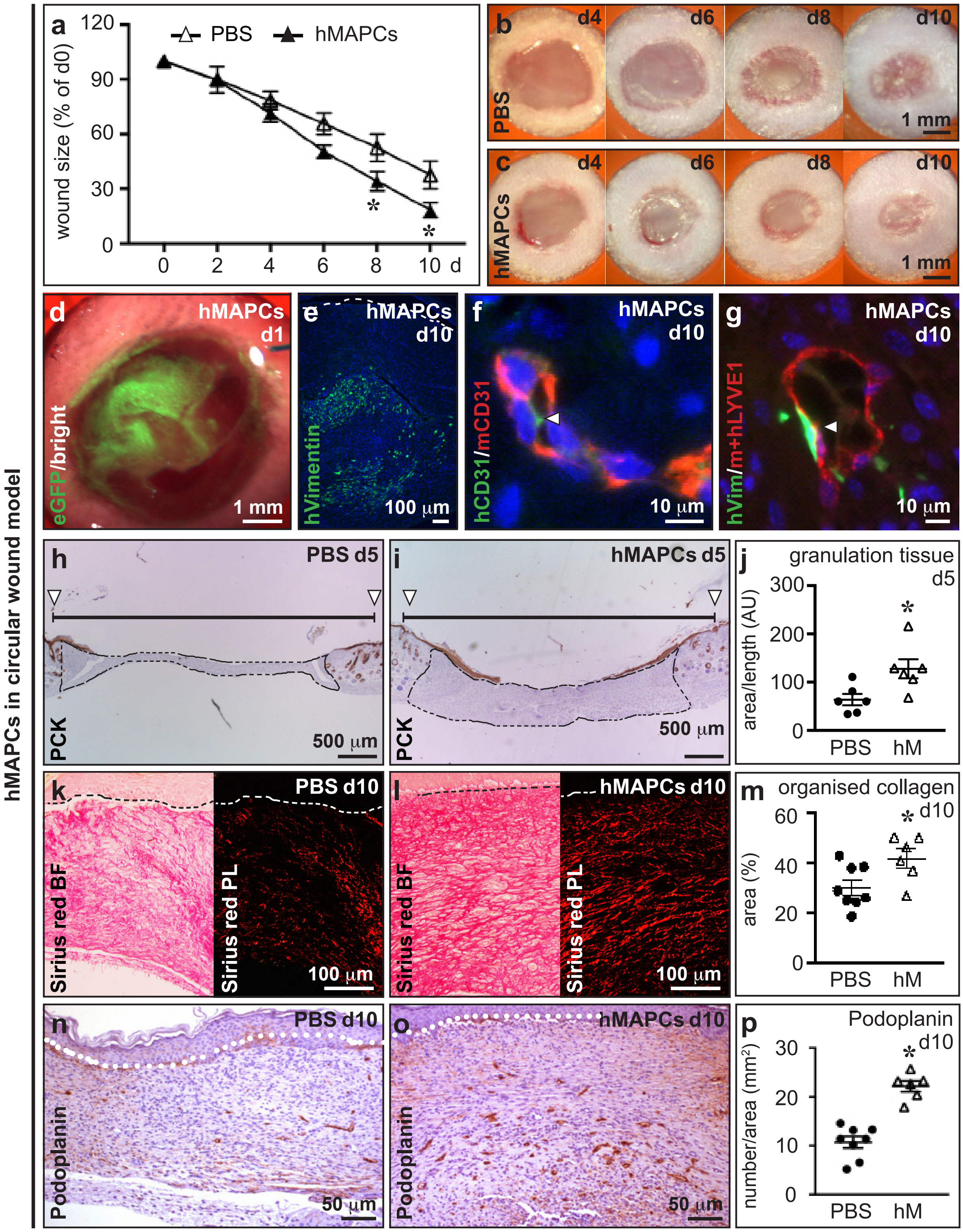

Supplementary Figure S4. hMAPCs promote wound healing. a-c, Diagram (*a*) representing wound size (in % *versus* size on day (d) 0) in mice treated with PBS (*n*=9: open triangles) or human (h)MAPCs (*n*=6; filled triangles) until 10 d after wounding and representative bright field pictures of the wounds on d4, d6, d8 and d10 after wounding of a PBS-treated (*b*) or hMAPC-treated (*c*) mouse. Quantitative data represent mean ± s.e.m. **P*<0.05 *versus* PBS by repeated measures ANOVA with Fisher post-hoc test. **d,** Merged picture of bright field and fluorescence image of the wound bed 24

hours after seeding of eGFP-labelled hMAPCs (in green) revealing homogenous distribution of $eGFP^+$ hMAPCs across the wound area. **e,** Image of a vimentin-stained (in green) wound cross-section of a mouse transplanted with hMAPCs 10 d earlier revealing persistence of large patches of hMAPCs homogenously distributed across the wound bed. The dermo-epidermal junction is indicated by a dashed line. **f,** Picture of a cross-section of a 10 d-old wound from a mouse treated with hMAPCs co-stained with anti-mouse (m)CD31 (in red) and anti-human (h)CD31 (in green), showing intercalation of a transplanted $CD31^+$ hMAPC (indicated by arrowhead) in a $CD31^+$ mouse vessel. **g,** Image of a wound cross-section of a mouse transplanted with hMAPCs 10 d earlier revealing occasional co-localisation (indicated by arrowhead) of hVimentin (in green) with LYVE1 (in red). **h-j,** Cross-sections stained for pancytokeratin (PCK) of PBS (*h*) or hMAPC-treated (*i*; 'hM') wounds and corresponding quantification (*j*) showing granulation tissue indicated by dashed black line in the wound area (delineated by open white triangles in *h*, *i* indicating the wound borders and by a black line indicating wound length). Data in *j* represent mean area of granulation tissue corrected for wound length expressed in arbitrary units (AU) ± s.e.m. n=6, P=0.02 *versus* PBS by unpaired two-tailed Student's t-test. **k-m,** Cross-sections stained for Sirius red (left panels represent bright field (BF), right panels represent corresponding polarised light (PL) images) of PBS (*k*) or hMAPC-treated (*l*; 'hM') 10 d-old wounds and corresponding quantification (*m*) showing organised collagen in red (the dermo-epidermal junction is indicated by a dashed line). Data in *m* represent mean area of organised collagen relative to the total amount of collagen expressed in % ± s.e.m. n=6-8, P=0.03 *versus* PBS by unpaired two-tailed Student's t-test. **n-p,** Podoplanin-stained (in brown) cross-sections of 10 d-old wounds treated with PBS (*n*) or hMAPCs (*o*), and corresponding quantification (*p*; expressed as number of vessels per area in mm^2; n=6-8; *P<0.0001 *versus* PBS by unpaired two-tailed Student's t-test). The dermo-epidermal junction is indicated by a dotted line. DAPI or TOPRO3 were used as nuclear counterstain in *e* or *f,g*, respectively. Haematoxylin was used as nuclear counterstain in *h,i,n,o*.

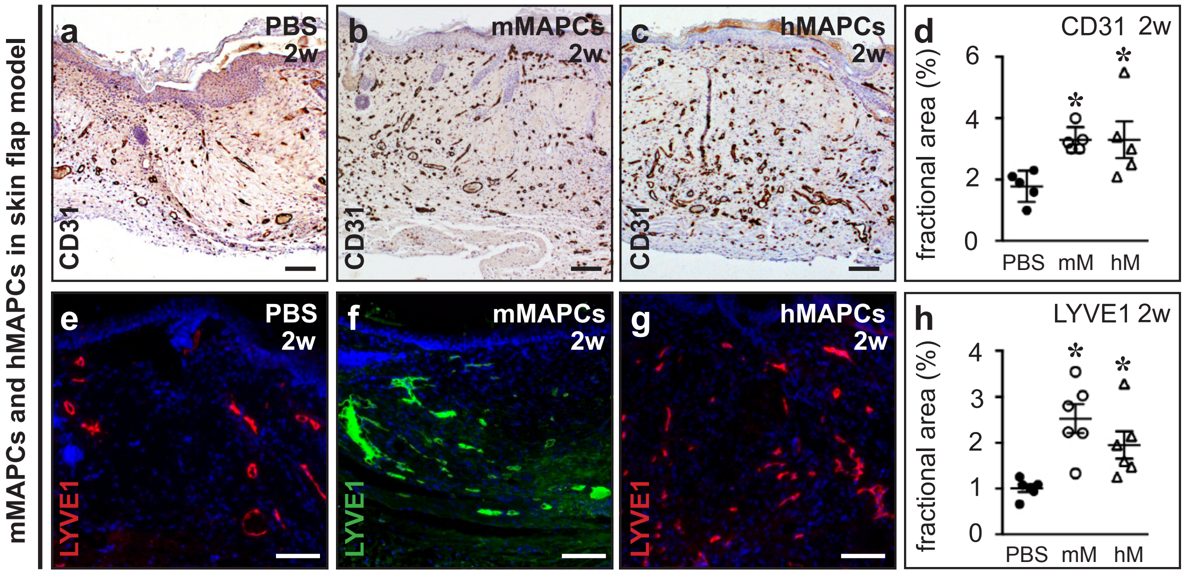

Supplementary Figure S5. MAPCs boost blood and lymphatic vessel growth in the skin flap model. a-d, Representative pictures of cross-sections of the skin wound (around the location of transplantation indicated by 'X' in Fig. 3a) from mice 2 weeks (w) after treatment with PBS (*a*), mMAPCs ('mM'; *b*) or hMAPCs ('hM'; *c*) stained for CD31, and corresponding quantification (*d*; data represent mean ± s.e.m. P=0.0053 by Kruskal-Wallis test; *P<0.05 *versus* PBS by Dunn's post-hoc test; n=5). **e-h,** Representative pictures of cross-sections of the skin wound (around the location of dextran injection indicated by arrow in Fig. 3a) from mice 2w after treatment with PBS (*e*), mMAPCs ('mM'; *f*) or hMAPCs ('hM'; *g*) stained for LYVE1 (red in *e*,*g*; green in *f*), and corresponding quantification (*h*; data represent mean ± s.e.m. P=0.0002 by Kruskal-Wallis test; *P<0.05 *versus* PBS by Dunn's post-hoc test; n=6).

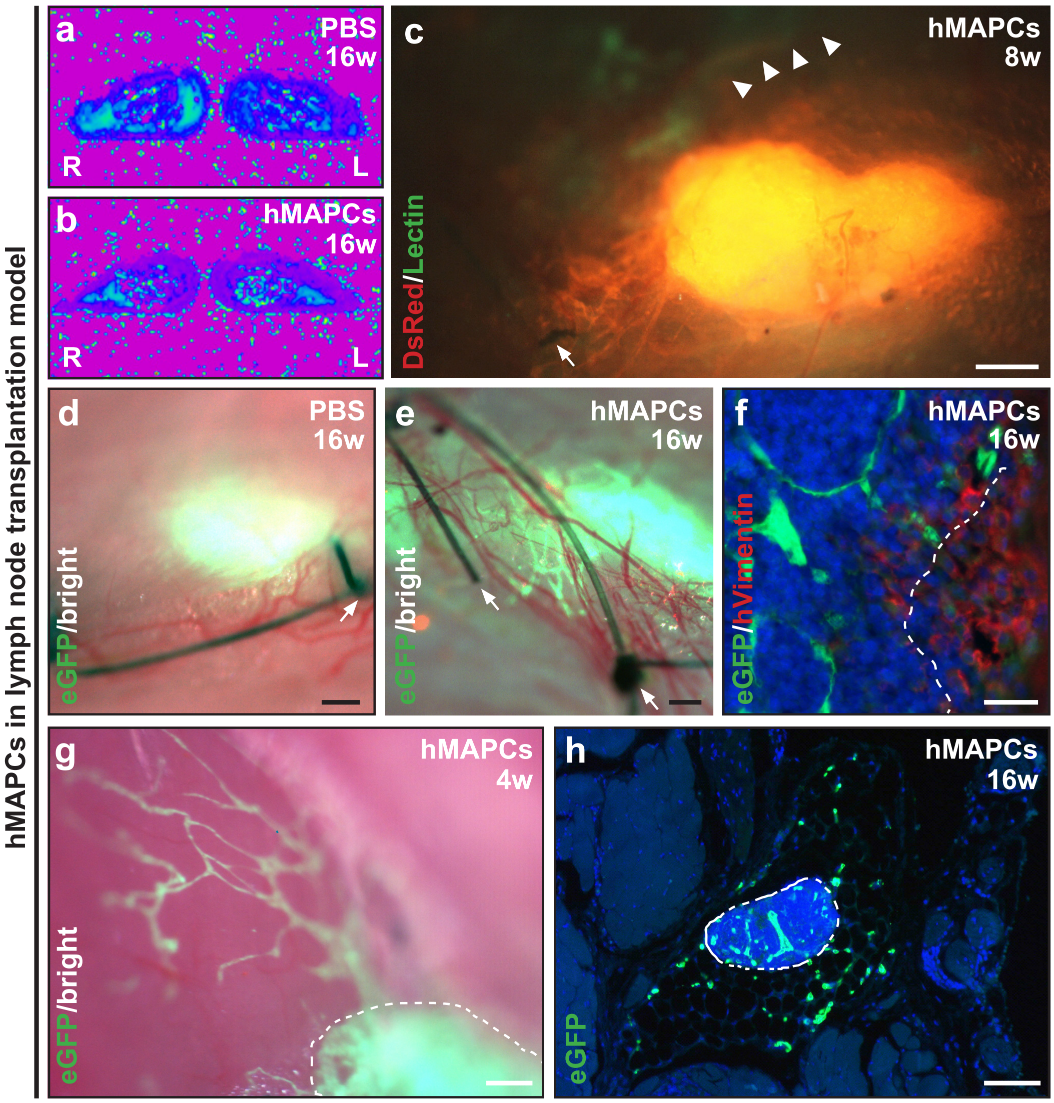

Supplementary Figure S6. hMAPCs support functional reconnection of transplanted lymph nodes to the host lymphatic network, as well as lymph node blood vessel supply and branching. a,b, T_2 maps corresponding to the T_2-weighted magnetic resonance (MR) images shown in Fig. 5c,d of the antebrachial regions of mice treated with Matrigel containing PBS (*a*) or hMAPCs (*b*), recorded 16 weeks (w) after lymph node transplantation. L: left; R: right. **c,** Merged picture of green and red fluorescence microscopic images of the right axillary region of a mouse transplanted with a DsRed$^+$ lymph node and treated with Matrigel containing hMAPCs 8w earlier. Note the connecting lymphatic vessel filled with FITC-labelled lectin (in green), indicated by arrowheads. **d,e,** Merged pictures of bright field and green fluorescence images of the right axillary region of mice transplanted with an eGFP$^+$ lymph node and treated with Matrigel containing PBS (*d*) or hMAPCs (*e*) 16w earlier, revealing a more elaborate blood vessel network irrigating the transplanted lymph node of hMAPC-treated mice. **f,** Merged picture of a red and green fluorescence image of a cross-section of the right axillary region of a mouse transplanted with an eGFP$^+$ lymph node and treated with hMAPCs 16w

earlier, revealing persisting vimentin-stained (in red) hMAPCs surrounding the transplanted lymph node. **g,** Merged picture of bright field and green fluorescence images of the right axillary region of a mouse transplanted with an $eGFP^+$ lymph node and treated with Matrigel containing hMAPCs 4w earlier, revealing outward branching of the (lymph)vascular network. **h,** Picture of an eGFP-stained cross-section of the right axillary region of a mouse transplanted with an $eGFP^+$ lymph node and treated with Matrigel containing hMAPCs 16w earlier, revealing distant outward branching of the (lymph)vascular network. Permanent sutures fixing the transplanted lymph node are indicated by arrows in *c-e*. Lymph node body is lined by a white dashed line in *f-h*. DAPI was used to reveal nuclei in *f,h*.

C. SUPPLEMENTARY TABLES

Supplementary Table S1. Antibody array coordinate maps

Mouse (ARY015)		Human (ARY007)	
**Coordinate**	**target/control**	**Coordinate**	**target/control**
	**Common**		
A1,A2,A21,A22,F1,F2	Reference spots	A1,A2,A23,A24,F1,F2	Reference spots
F19, F20	Negative control	F23,F24	Negative control
A5,A6	ADAMTS-1	A7,A8	ADAMTS-1
A7,A8	Amphiregulin	A17,A18	Amphiregulin
A9,A10	Angiogenin	A9,A10	Angiogenin
A11,A12	ANG-1	A11,A12	ANG-1
A17,A18	CXCL16	B3,B4	CXCL16
B7,B8	DPPIV	B5,B6	DPPIV
B9,B10	EGF	B7,B8	EGF
B11,B12	Endoglin	B11,B12	Endoglin
B13,B14	Endostatin	B13,B14	Endostatin
B15,B16	Endothelin-1	B15,B16	Endothelin-1
B17,B18	FGF-1	B17,B18	FGF-1
B19,B20	FGF-2	B19,B20	FGF-2
C3,C4	FGF-7/KGF	B23,B24	FGF-7/KGF
C7,C8	GM-CSF	C3,C4	GM-CSF
C9,C10	HB-EGF	C5,C6	HB-EGF
C11,C12	HGF	C7,C8	HGF
C13,C14	IGFBP-1	C9,C10	IGFBP-1
C15,C16	IGFBP-2	C11,C12	IGFBP-2
C17,C18	IGFBP-3	C13,C14	IGFBP-3
C21,C22	IL-1beta	C15,C16	IL-1beta
D9,D10	Leptin	C21,C22	Leptin
D11,D12	MCP-1	C23,C24	MCP-1
D13,D14	MIP-1alpha	D1,D2	MIP-1alpha
D17,D18	MMP-8	D3,D4	MMP-8
D19,D20	MMP-9	D5,D6	MMP-9
F5,F6	PAI-1	E3,E4	PAI-1
E5,E6	PD-ECGF	D11,D12	PD-ECGF
E7,E8	PDGF-AA	D13,D14	PDGF-AA
E9,E10	PDGF-AB/BB	D15,D16	PDGF-AB/BB
F7,F8	PEDF	E5,E6	PEDF
E11,E12	Pentraxin3	D9,D10	Pentraxin3
E13,E14	PF4	D19,D20	PF4
E15,E16	PLGF-2	D21,D22	PLGF
E17,E18	Prolactin	D23,D24	Prolactin
A15,A16	TF	B1,B2	TF
F11,F12	TIMP-1	E7,E8	TIMP-1
F13,F14	TIMP-4	E9,E10	TIMP-4
F9,F10	TSP-2	E13,E14	TSP-2
F15,F16	VEGF-A	E19,E20	VEGF-A

Mouse (ARY015)		Human (ARY007)	
**Coordinate**	**target/control**	**Coordinate**	**target/control**
**Not common**			
A13,A14	ANG-3	A5,A6	Activin-A
B3,B4	CYR61	A13,A14	ANG-2
B5,B6	DLL4	A15,A16	Angiostatin
C5,C6	Fractalkine	A19,A20	Artemin
C19,C20	IL-1alpha	B9,B10	EG-VEGF
D3,D4	IL-10	B21,B22	FGF-4
D5,D6	IP-10	C1,C2	GDNF
D7,D8	CXCL1	C17,C18	IL-8
D15,D16	MMP-3	C19,C20	TGF-beta1
D21,D22	NOV	D7,D8	NRG1-beta1
E3,E4	Osteopontin	D17,D18	Persephin
E19,E20	Proliferin	E1,E2	Serpin-B5
F3,F4	SDF-1	E11,E12	TSP-1
F17,F18	VEGF-B	E15,E16	uPA
-	-	E17,E18	Vasohibin
-	-	E21,E22	VEGF-C

Abbreviations: ADAMTS-1: A Disintegrin and Metalloproteinase with Thrombospondin motifs-1; ANG: angiopoietin; DPPIV: dipeptidyl peptidase-4; EGF: epidermal growth factor; FGF: fibroblast growth factor; KGF: keratinocyte growth factor; GM-CSF: granulocyte macrophage-colony stimulating factor; HB-EGF: heparin binding EGF-like growth factor; HGF: hepatocyte growth factor; IGFBP: insulin growth factor binding protein; IL: interleukin; MCP-1: monocyte/macrophage chemoattractant protein-1; MIP: macrophage inflammatory protein; MMP: matrix metalloproteinase; PAI-1: plasminogen activator inhibitor-1; PD-ECGF: platelet-derived endothelial cell growth factor; PDGF: platelet-derived growth factor; PEDF: pigment epithelium-derived factor; PF: platelet factor; PLGF: placental growth factor; TF: tissue factor; TIMP: tissue inhibitor of matrix metalloproteinases; TSP: thrombospondin; VEGF: vascular endothelial growth factor; CYR61: cystein-rich angiogenic inducer 61; DLL4: delta-like 4; IP: interferon-gamma-inducible protein; NOV: nephroblastoma overexpressed; SDF-1: stromal cell-derived factor-1; EG-VEGF: endocrine gland-vascular endothelial growth factor; GDNF: glial cell-derived neurotrophic factor; TGF: transforming growth factor; NRG1: neuregulin-1; uPA: urokinase-type plasminogen activator.

Supplementary Table S2. List of antibodies for histology or cell staining

Antigen	Target species	Supplier, catalog N°
CD31	mouse	Beckton Dickinson, 557355
CD31	human	DAKO, M0823
LYVE1	mouse + human	Upstate Biotechnology, 07-538
Pancytokeratin (PCK)	mouse	Sigma, C-2562
Podoplanin	mouse	eBioscience, 14-5381
Flt4	mouse	eBioscience, 14-5988-82
Smooth muscle α-actin (SMA)	mouse + human	Sigma C-6148 or A5228
CD45	mouse	Beckton Dickinson, 553076
Prox1	mouse + human	Angiobio, 11-002
Vimentin	human	DAKO, Clone V9
eGFP	-	Abcam, ab13970
Ki67	mouse + human	Abcam, ab15580

Supplementary Table S3. qRT-PCR primers

Gene	5'-3' forward primer	5'-3' reverse primer
Prox1 (M)	*CGCGTGGGTTTCTTCTCTGC*	*GGGCTGTGCTGTCATGGTCA*
Pdpn (M)	*GCCAGTGTTGTTCTGGGTTT*	*AGAGGTGCCTTGCCAGTAGA*
Itga9 (M)	*CTGCTTTCCAGTGTTGACGA*	*AATGCCCATCTCCTCCTTCT*
Flt1 (M)	*TGGCCAGAGGCATGGAGT*	*TCGCAAATCTTCACCACATGG*
Tek1 (M)	*GAAACATCCCTCACCTGCAT*	*TGGCCTTTTCTCTCTTCCAA*
vWF (M)	*AAGGAGCAGGACCTGGAAGT*	*GCGTGTATGTGAGGATGTGG*
Gapdh (M)	*CCGCATCTTCTTGTGCAGT*	*GAATTTGCCGTGAGTGGAGT*
PROX1 (H)	*CAGTACTGAAGAGCTGTCTATAACCAGAG*	*TCTGAGCAACTTCCAGGAATCTC*
PDPN (H)	*TGCTCTTCGTTTTGGGAAGC*	*TCGCTGGTTCCTGGAGTCAC*
ITGA9 (H)	*AGGACGCTGATCCCTTGCTA*	*GCACTTTGATGGTTCCAGCC*
FLT1 (H)	*TTTGGATGAGCAGTGTGAGC*	*CGGCACGTAGGTGATTTCTT*
TEK1 (H)	*ACACCTGCCTCATGCTCAGC*	*AGCAGTACAGAGATGGTTGCATTC*
VWF (H)	*TGCTGGTATGGAGTATAGGCAGTG*	*CCGGAATGCACGCAGG*
VEGF-A (H)	*ACCAAGGCCAGCACATAGGA*	*AGGCCCACAGGGATTTTCTT*
VEGF-C (H)	*CTCTCTCTCAAGGCCCCAAA*	*TTGTTCGCTGCCTGACACTG*
ANG-2 (H)	*TGGAAGACAAGCACATCATC*	*CAACAGTGGGGTCCTTAGCT*
GAPDH (H)	*TGGTATCGTGGAAGGACTCATGAC*	*ATGCCAGTGAGCTTCCCGTTCAGC*

M: mouse; H: human.

D. REFERENCES IN SUPPLEMENT

1. Aranguren, X. L. *et al*. Multipotent adult progenitor cells sustain function of ischemic limbs in mice. *J Clin Invest* **118**, 505-514 (2008).
2. Ulloa-Montoya, F. *et al*. Comparative transcriptome analysis of embryonic and adult stem cells with extended and limited differentiation capacity. *Genome Biol* **8**, R163 (2007).
3. Aranguren, X. L. *et al*. In vitro and in vivo arterial differentiation of human multipotent adult progenitor cells. *Blood* **109**, 2634-2642 (2007).
4. Roobrouck, V. D. *et al*. Differentiation potential of human postnatal mesenchymal stem cells, mesoangioblasts, and multipotent adult progenitor cells reflected in their transcriptome and partially influenced by the culture conditions. *Stem Cells* **29**, 871-882 (2011).
5. Aranguren, X. L. *et al*. COUP-TFII orchestrates venous and lymphatic endothelial identity by homo- or hetero-dimerisation with PROX1. *J Cell Sci* **126**, 1164-1175 (2013).
6. Seaman, W. E., Sleisenger, M., Eriksson, E. & Koo, G. C. Depletion of natural killer cells in mice by monoclonal antibody to NK-1.1. Reduction in host defense against malignancy without loss of cellular or humoral immunity. *J Immunol* **138**, 4539-4544 (1987).
7. Saaristo, A. *et al*. Vascular endothelial growth factor-C gene therapy restores lymphatic flow across incision wounds. *FASEB J* **18**, 1707-1709, doi:10.1096/fj.04-1592fje (2004).
8. Tammela, T. *et al*. Therapeutic differentiation and maturation of lymphatic vessels after lymph node dissection and transplantation. *Nat Med* **13**, 1458-1466 (2007).
9. Hendrickx, B. *et al*. Integration of blood outgrowth endothelial cells in dermal fibroblast sheets promotes full thickness wound healing. *Stem Cells* **28**, 1165-1177 (2010).
